# Single‐Cell Transcriptome Landscape and Cell Fate Decoding in Human Brain Organoids after Transplantation

**DOI:** 10.1002/advs.202402287

**Published:** 2024-05-06

**Authors:** Shi‐Bo Xu, Xin‐Rui Li, Pan Fan, Xiyang Li, Yuan Hong, Xiao Han, Shanshan Wu, Chu Chu, Yuejun Chen, Min Xu, Mingyan Lin, Xing Guo, Yan Liu

**Affiliations:** ^1^ State Key Laboratory of Reproductive Medicine Institute for Stem Cell and Neural Regeneration School of Pharmacy Key Laboratory of Targeted Intervention of Cardiovascular Disease Collaborative Innovation Center for Cardiovascular Disease Translational Medicine Nanjing Medical University Nanjing 211166 P. R. China; ^2^ State Key Laboratory of Reproductive Medicine Department of Neurobiology School of Basic Medical Sciences Nanjing Medical University Nanjing 211166 P. R. China; ^3^ Institute of Neuroscience Key Laboratory of Primate Neurobiology CAS Center for Excellence in Brain Science and Intelligence Technology Chinese Academy of Sciences Shanghai 200031 China; ^4^ Co‐innovation Center of Neuroregeneration Nantong University Jiangsu 226019 China

**Keywords:** astrocyte, human brain organoids, neurotransmitter, single‐cell RNA sequencing, transplantation therapy

## Abstract

Human stem cells and derivatives transplantation are widely used to treat nervous system diseases, while the fate determination of transplanted cells is not well elucidated. To explore cell fate changes of human brain organoids before and after transplantation, human brain organoids are transplanted into prefrontal cortex (PFC) and hippocampus (HIP), respectively. Single‐cell sequencing is then performed. According to time‐series sample comparison, transplanted cells mainly undergo neural development at 2 months post‐transplantation (MPT) and then glial development at 4MPT, respectively. A different brain region sample comparison shows that organoids grafted to PFC have obtained cell fate close to those of host cells in PFC, other than HIP, which may be regulated by the abundant expression of dopamine (DA) and acetylcholine (Ach) in PFC. Meanwhile, morphological complexity of human astrocyte grafts is greater in PFC than in HIP. DA and Ach both activate the calcium activity and increase morphological complexity of astrocytes in vitro. This study demonstrates that human brain organoids receive host niche factor regulation after transplantation, resulting in the alignment of grafted cell fate with implanted brain regions, which may contribute to a better understanding of cell transplantation and regenerative medicine.

## Graphical abstract

Grafted human brain organoids in the host brain keep forebrain cell fate following intrinsic expression patterns similar to fetal brain tissue. Grafted organoids undergo neural and astrocytic differentiation successively. Young human cells acquire properties similar to the brain regions after grafting. Neurotransmitters enhance the activity and morphological complexity of astrocytes in organoids.

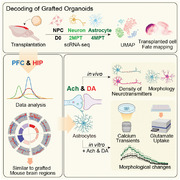

## 1 Introduction

The adult central nervous system (CNS) has limited self‐repair capacity after pathogenesis or tissue impair, while young cells, which are equiv. to neurons in embryonic stage, or tissue replacement may be an effective approach to repair brain. Neural progenitor cells derived from human pluripotent stem cells could be considered as a young cell pool for cell repairing in CNS. Recently, several transplantation therapies using human stem cells and derivatives have been applied in the clinic, providing hope for neural repairment.^[^ ^1^, ^2^ ^]^

Recently, brain organoids have been demonstrated to be promising cell donors for repairing severe neural injury at tissue level.^[^ ^3^, ^4^ ^]^ And transplanted human organoids have been proven to integrate into the recipient's neural circuit and coordinate with their behaviors. For example, the host vessels were observed to progressively infiltrate into human organoids after the transplantation of organoids into unilaterally injured mouse brains.^[^ ^5^ ^]^ Neurons in the implanted organoids furthermore presented more complex morphology, synapses, and membrane properties than that in vitro.^[^ ^6^ ^]^ Our previous work also demonstrated that grafted human brain organoids present long projections to the deep brain region and establish functional connections with the host neurons.^[^ ^7^ ^]^

Before translational cell‐based therapy, fate determination of the grafted organoids, as well as safety analysis, need to be comprehensively evaluated. In vitro, time‐series studies for the cell fate determination of human brain organoids have been described;^[^ ^8^ ^]^ In vivo, numerous studies, including ours, have been devoted to study the survival, neural circuit integration, and functional recovery after transplantation of human pluripotent stem cells (hPSCs)‐derived neurons differentiation, but how the cell fate determination of the grafted hPSC‐derived neuron or organoid is still unknown. Therefore, it is essential to perform dual cell fate decoding of time and space for transplanted neural cells, and the interactive regulation of their own genes and the host micro‐environment (or niche) should be fully explored.

Here, we transplanted human brain organoids into PFC or HIP of mice and obtained more than 30 000 high‐quality human‐derived cells. Transplanted cells mainly underwent neural development at 2 months post‐transplantation (MPT) and then glial proliferation at 4MPT, respectively. In different brain‐region comparisons, we found human cells acquired the transcriptomic properties of grafted host brain region. Furthermore, astrocytes transplanted in PFC were more mature than those in HIP, which was enhanced by receiving rich neurotransmitters, dopamine (DA) and acetylcholine (Ach). Our results may pave the road for applications of human brain organoids into transplantation therapy.

## 2 Results

### 2.1 Single‐Cell Transcriptome of Grafted Human Brain Organoids Recapitulates Developing Fetal Forebrain Features

**Figure 1:**
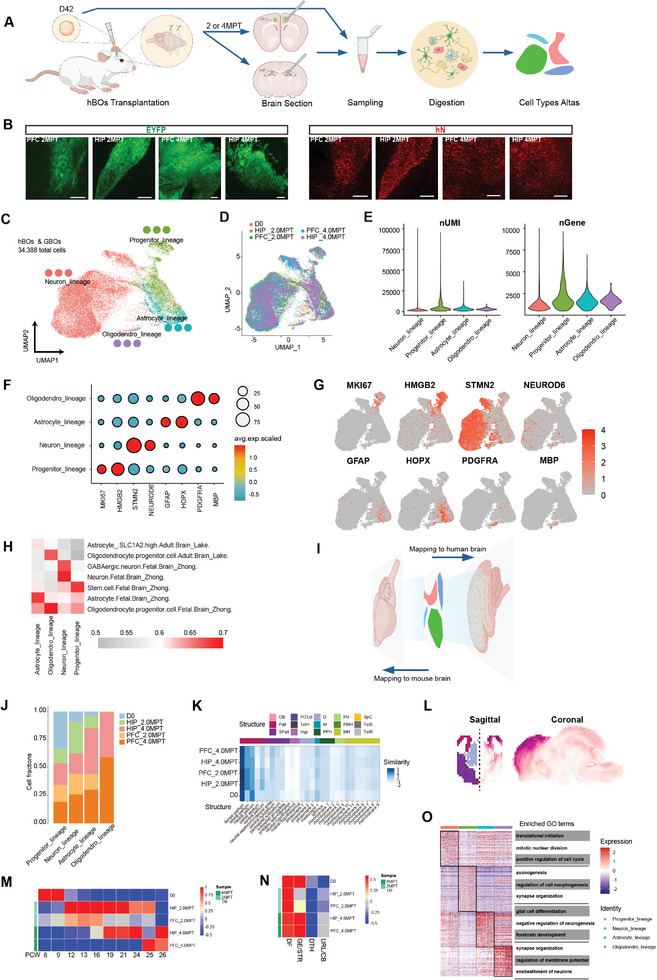
Single‐cell Transcriptome of Grafted Human Brain Organoids Recapitulates Developing Fetal Forebrain Features. A) Schematic diagram showing the processes of human brain organoid transplantation, sampling, digestion, sequencing, and analysis. B) Representative immunofluorescence results after human brain organoid transplantation. HIP_2MPT, HIP_4MPT represents the organoids at 2 and 4 months after transplantation to the mouse HIP, respectively; PFC_2MPT and PFC_4MPT: the organoids at 2 and 4 months after transplantation to the mouse PFC, respectively. Exogenous cell marker: EYFP; human‐derived nuclear marker: hN. Scale bar: 100 µm. C) UMAP visualization of the distribution of human cells in organoids before and after transplantation, with colored dots marking cell types. hBOs: human brain organoids; GBOs: grafted human brain organoids (n = 34388 cells from 20 organoids in vitro and 16 grafted mice in vivo). D) UMAP plot between different samples. D0: organoids acquired on the day of transplantation. E) Violin plots revealing differences in numbers of expressed genes and transcripts among types of cells. F) Dot plot showing the expression of characteristic genes of different cell types. The diameter of the dot represents the proportion of expressing cells; the colors show the normalized gene expression. G) The expression of characteristic genes (same as F) of the four major cell types in hBOs and GBOs. H) Heat map presenting the correlation between cells in our data and human brain cells in the scHCL database. Using R package scHCL. I) Mapping the scRNA‐seq data onto the human brain or mouse brain public database. J) Bar chart: the length of a bar shows the number of cells. Columns represent cell types; colors represent samples. K) The correlation between each sample and E13.5 mouse brain regions from Allen Brain Institute Database. L) VoxHunt showing the most similar brain region (E13.5 mouse brain sections) to which organoids were mapped. Left: coronal section; right: sagittal section. Reference data is from Allen Brain Institute Database. M) Heatmap presenting the correlation between each sample and human fetal brains from the BrainSpan database at different stages of development. N) The most similar region of a human organoid compared with human embryonic brain section. DF; ventral striatum (GE/STR); thalamus (DTH); cerebellum (URL/CB). O) Heatmap showing gene sets specifically expressed by each cell lineage and the corresponding enriched GO terms.

To explore cell fate of human brain organoids after transplantation, the cell transcriptomic profiling of human organoids was determined by single‐cell RNA sequencing, with the comparison of brain organoids before transplantation. Human brain organoids derived from H9: AAVS‐ChR2‐EYFP^[^ ^9^ ^]^ labeled with EYFP fluorescent protein were implanted into the prefrontal cortex (PFC) and hippocampus (HIP) of a mouse (SCID‐Beige) brain, respectively. Then single‐cell sequencing was performed for grafted brain organoids (GBOs) in the mouse brain at 2 months post transplantation (MPT) or 4MPT (**Figure** **1**A).

Following a previously established protocol to differentiate human brain organoids in vitro,^[^ ^7^ ^]^ D42 human brain organoids were generated (Figure S1A,B, Supporting Information). Then human brain organoids were bilaterally transplanted into PFC or HIP of mice, respectively. Grafted human cells were distinguished by EYFP or hN (Figure 1B; Figure S1C, Supporting Information). Several blood vessels were found to integrate into grafted organoids within 1MPT (Figure S1E, Supporting Information), and human synaptic labeling hSYN was determined on human fibers near the implants (Figure S1F, Supporting Information), which suggested that grafted organoids survived and formed synaptic connections to host brain. In addition, nearly no pluripotent cell marker NANOG was detected in grafted organoids (Figures S1D and S2K, Supporting Information). Furthermore, no teratoma or malignant proliferation was found in transplanted animals (n > 70 mice). These results indicated that organoids transplantation has a low risk for tumor genesis in our system.

Single‐cell suspensions were prepared from five groups, including D0 (day 42 brain organoids that pre‐transplantation), PFC_2MPT, PFC_4MPT, HIP_2MPT, and HIP_4MPT. A total of 34388 human cells were obtained from 16 grafted host brains and ≈20 human brain organoids after quality control (Figure S2F–I, Supporting Information). On average, 2262 transcripts and 1232 genes were generated for each cell.

Given that the mixing of murine cells during acquisition of grafted organoids was inevitable, host cells were excluded to purify the grafted human datasheet during analysis. First, after reads were mapped to human‐mouse dual reference genome, ratios of human transcripts were calculated for each cell (Figure S2A, Supporting Information). The results showed two aggregation peaks (low or high) which suggested clusters of murine or human cells, respectively. Then all the cells were divided into different species identities by two ratios (0.2 and 0.8), and the cells in between were tentatively labeled as Unknown Cells. It is evident that three cell groups showed their own distribution in scatter plot, which may suggest the reliability of labeling (Figure S2B, Supporting Information).

The Uniform Manifold Approximation and Projection (UMAP) analysis was performed to investigate the diversity of human cells using Seurat. All the cells were unbiasedly isolated into four major cell types by global transcriptomes, such as, progenitor_lineage (PLC), neuron_lineage (NLC), astrocyte_lineage (ALC), and oligodendro_lineage (OLC) (Figure 1C,D). In total, four major cell types were identified in human cells according to the expression of specific genes, including PLC (MI67, HMGB2), NLC (STMN2, NEUROD6), ALC (GFAP, HOPX), and OLC (PDFGRA, MBP) (Figure 1F,G). Meanwhile, both GO enrichment analysis and comparison of our data and human brain tissue transcriptome data from SCHCL^[^ ^10^ ^]^ confirmed the validity of classification (Figure 1H,O). The proportions of the four types of cells varied among the five groups. Specifically, group D0 showed the highest proportion in progenitor cells (33.4%). The population of neurons was notably increased after transplantation (D0: 9.5% vs After‐graft: average 22.6%), which was similar to astrocytes (D0: 4.1% vs After‐graft: average 24.0%). Oligodendrocytes appeared only in 4MPT groups (Figure 1J), indicating that organoids matured after grafting. Although human‐derived oligodendrocytes were a small population, they were identified by co‐labeling human cytoplasmic marker STEM121 with oligodendrocyte marker MBP in brain slices (Figure S1G, Supporting Information). Furthermore, human cells were unbiasedly subclassified and more cell subtypes were found, such as cycling progenitors, radial glia, and intermediate progenitors et al (Figure S2L,M, Supporting Information). Then their identities were also confirmed by characteristic genes (Figure S2M–Q, Supporting Information).

To assess the brain regional identity of grafted cells, all samples were mapped onto embryonic day 13.5 (E13.5) mouse brain data from Allen Brain Institute.^[^ ^11^ ^]^ Both organoids and grafted organoids were found to mostly map onto dorsal telencephalon (Figure 1K). And VoxHunt algorithm supported the above conclusion in both coronal and sagittal section view (Figure 1L). In addition, human organoids were compared with human fetal brain data from BrainSpan, and found all of them showed a strong correlation with dorsal forebrain (DF) (Figure 1N). According to the developmental period, all samples could be separated into three stages: group D0 was close to 8–9 w human fetal brain, group 2MPT was similar to 12–16 w of gestation, and group 4MPT was close to 19–26 w of gestation (Figure 1M). These results suggested that grafted human brain organoids undergo a progressive development and maturation.

Taken together, the single cell transcriptome of human brain organoids was acquired after transplantation. All the human cells were divided into four major cell types and grafted organoids maintained their forebrain cell fate after transplantation.

### 2.2 Human Organoids Undergo Neural Differentiation and Astrocytic Differentiation Successively after Transplantation

**Figure 2:**
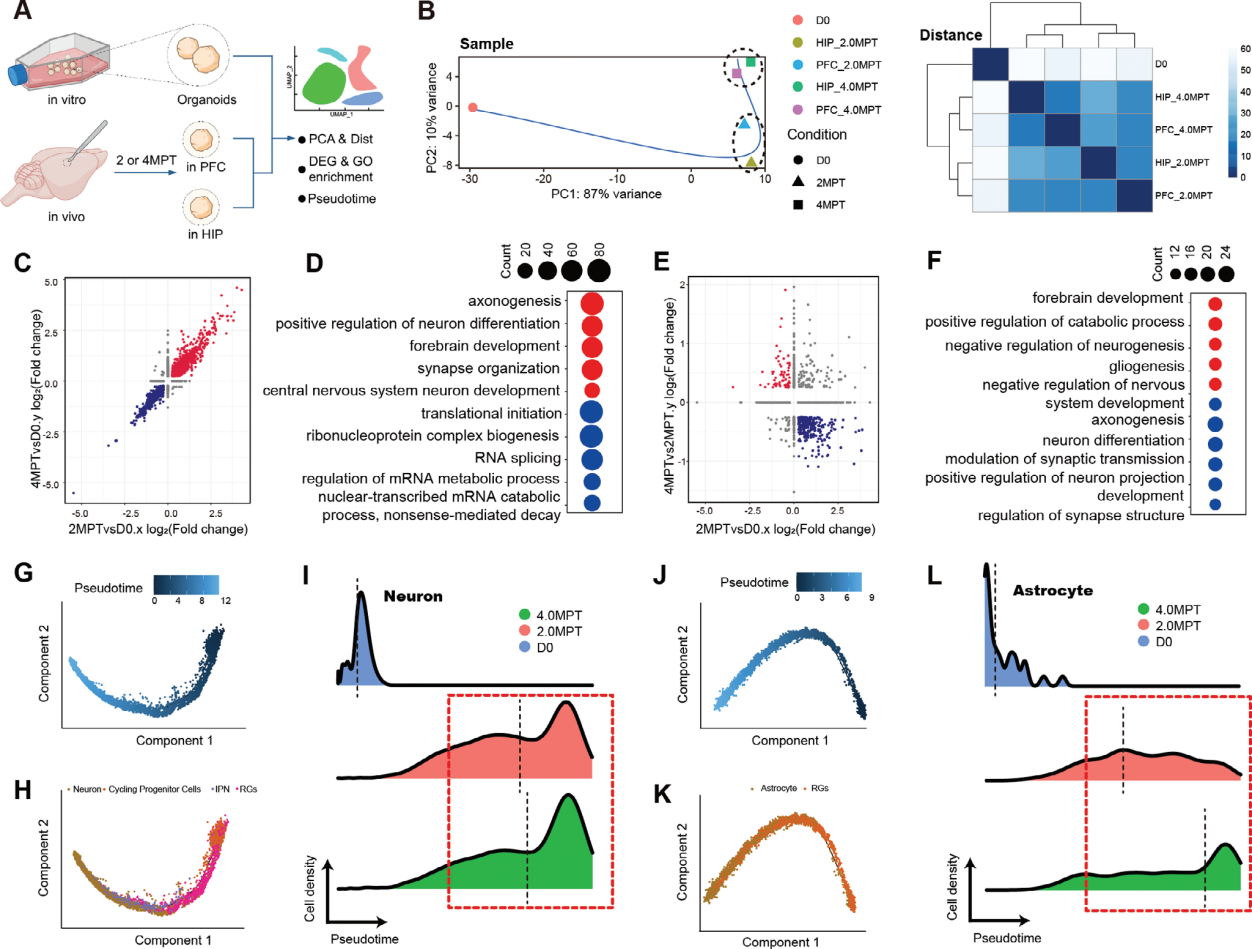
Human Organoids Undergo Neuronal and Glial Differentiation in the Second and Fourth Months Individually after Transplantation. A) Illustration showing the difference analysis of the single cell sample of each group. B) (left) Principal component analysis showing the difference in pseudo‐bulk RNA expression among the five groups of samples. Color: different samples, shape: different groups, and curve: the simulated developmental curve of groups. (right) Heat map describing the similarity between samples. Samples are clustered unbiasedly. the heatmap color represents the distance of transcriptome expression profiles among samples. C,E) Distribution scatterplot of DEGs. Dots represent the log_2_‐transformed fold change of gene expression in different DEGs. All red dots include genes in the first quadrant (in C) or the second quadrant (in E), respectively; all blue dots include genes in the third quadrant (in C) or the fourth quadrant (in E). D,F) Top terms after the enrichment analysis of GO biological process. Red or blue dots correspond to the gene sets in red or blue respectively (in C and E). The diameter of the dot represents the number of enriched genes. G,J) Single cell trajectories of different cell lineages (G: neurons, J: astrocytes). Pseudo‐time is indicated by the color of dot. H,K) Pseudo‐time trajectories recovered by Monocle. Colors represent cell subtypes. The distribution of cells along the trajectories reveals the order of cell development. During neuron development, CPCs, RGs, IPNs, and GNs appeared in the trajectory successively, and during astrocyte development, RGs were developed into Astrocytes. I,L) Pseudo‐time distribution of human cells. Colors represent sample groups. Black dashed lines represent the pseudo‐time averages of different groups.

To analyze the transcriptomic features among five samples, the single cell pseudo‐bulk mRNA expression of each sample was first obtained. Group D0 was found to be isolated from the other four groups in Principal Component Analysis (PCA), which indicated a substantial difference in transcriptome between pregraft organoids and postgraft. Furthermore, group 2MPT (HIP_2MPT and PFC_2MPT) was separated from group 4MPT (HIP_4MPT and PFC_4MPT), which showed that the diversity of samples in transcriptome after transplantation were mainly due to the experienced time of organoids. In addition, it leads to the same conclusion when the transcriptome‐based distance between groups and then clustered samples was calculated unbiasedly (**Figure** **2**B).

The differentially expressed genes (DEG) between groups were acquired for further analysis. There was a strong linear correlation between the log_2_(Fold change) of DEG of group 2MPT and group D0 and those of group 4MPT and group 2MPT (Figure 2C), which proved their DEG sets were similar. The coupregulated gene set (red spots in 2C) was selected for gene ontology (GO) biological process enrichment analysis, and nearly all the top enriched terms were related to neural development and maturation, such as axonogenesis, neuron differentiation, forebrain development, synapse organization, and neuron development. While codownregulated gene set (blue spots in 2C) enriched for metabolism and progenitor development, for example, translational initiation, ribonucleoprotein complex biogenesis, mRNA metabolic process, and nuclear‐transcribed mRNA catabolism (Figure 2D). These results suggested that human organoids were still in the early stage of development before transplantation and then underwent neural development, differentiation, and maturation after transplantation. In order to explore the reasons for the enormous transcriptomic differences between organoids before and after grafting, gene sets specially belong to group 2MPT and group D0 were further investigated, individually. The gene set 2MPT was enriched for neuron migration, neural development, and dendrite development, while gene set D0 was associated with hypoxia, cell stress, cellular respiration, and nuclear division (Figure S3C,D, Supporting Information). Stress‐related genes (HIF1A, VEGFA, ALKBH5: response to hypoxia;^[^ ^12^, ^13^, ^14^ ^]^ ARCN1, GORASP2, ATF6B, HSPA5: endoplasmic reticulum stress response;^[^ ^15^, ^16^ ^]^ PGK1: glycolysis gene^[^ ^16^ ^]^) were high expressed in group D0 (Figure S3E, Supporting Information) but decreased in group 2MPT and 4MPT, which is also consistent with the previous research.^[^ ^16^ ^]^ To eliminate temporal interference, a sample of human brain organoids cultured in vitro for ≈100 days was also compared with our grafted samples at 2MPT. The results reaffirmed that transplantation primarily led to the maturation of neurons and alleviation of stress response (Figure S3L–O, Supporting Information).

The log_2_(Fold change) of DEG between 4MPT and 2MPT was similarly compared with those between 2MPT and D0 (Figure 2E), and the genes (red spots) upregulated in 4MPT and downregulated in 2MPT were focused on. GO enrichment analysis showed these genes were predominantly associated with positive regulation of gliogenesis and negative regulation of neurogenesis (Figure 2F). In contrast, all the genes (blue spots) upregulated in 2MPT and downregulated in 4MPT were related to neural development and maturation (10/top 10, data not shown). Therefore, human organoids mainly went through neuron development in 2MPT and then glial genesis in 4MPT. In addition, genes upregulated both in 2MPT and 4MPT were enriched for neuron development and maturation (7/top 10) and genes downregulated were related to progenitor cell proliferation and development (Figure S3A,B, Supporting Information), which indicated human organoids mainly underwent neural development and maturation after transplantation.

To determine the pseudo‐time distribution of each group, single cell trajectories for all the human cells were reconstructed and three main developmental trajectory branches were found, for instance, cycling progenitor cells developed to radial glial cells and then to intermediate progenitor neurons which finally developed to glutamatergic neurons, radial glial cells developed to astrocytes, and oligodendrocyte progenitor cells developed to oligodendrocytes (Figure S3J,K, Supporting Information). To further assess the cell lineage differentiation, single‐cell branching trajectory analysis was performed for two main cell types, neurons and astrocytes. Cycling progenitor cells, radial glial cells, intermediate progenitor neurons, and glutamatergic neurons appeared in the neural trajectory successively, while radial glial cells and astrocytes appeared in astrocyte trajectory (Figure 2G–H,J,K). The results showed grafted neurons developed over time during the first two months and showed limited change in 4MPT (pseudo‐time averages: 2MPT:13.40 vs 4MPT:13.66). By contrast, astrocytes were more mature in 4MPT than in 2MPT (pseudo‐time averages: 2MPT:6.99 vs 4MPT:8.68) (Figure 2I,L). These results suggested that the grafted organoids developed into neurons at the beginning and then developed into astrocytes later, which demonstrated the transplanted organoids matured from 2MPT to 4MPT (Figure 2E,F).

**Figure 3:**
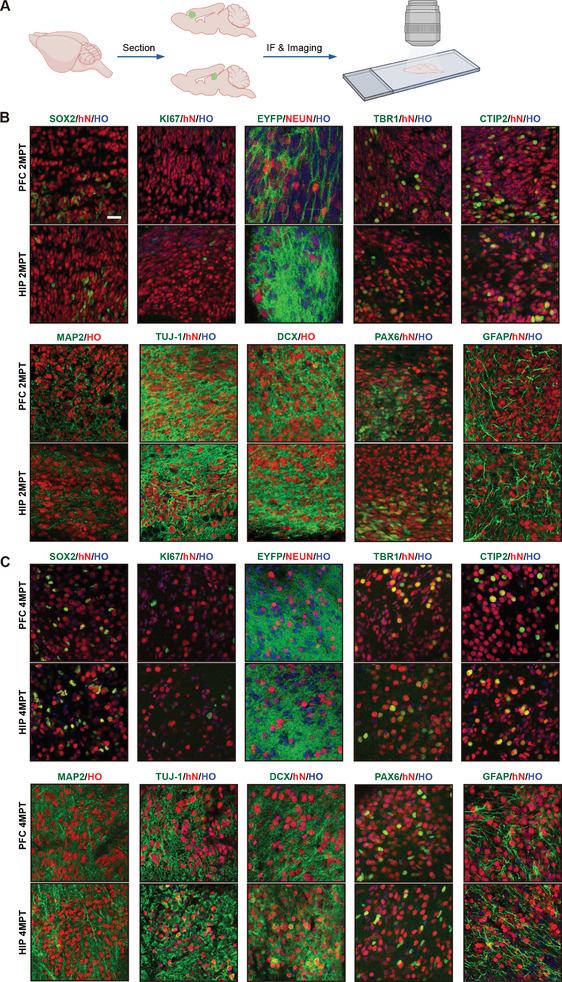
Immunofluorescence Results Reveal that Human Brain Organoids Mainly Undergo Astrocyte Differentiation after Transplantation. A) The immune fluorescent comparison of the human brain organs after transplantation into mouse HIP and PFC. B) Immunostaining results of molecular signatures of organoids in 2MPT. HO: labeling nucleus; hN: human nucleus‐associated protein; SOX2: labeling neural precursor cells; EYFP: enhanced yellow fluorescent protein; NEUN: expressed in mature neuron nuclei; TBR1 and CTIP2: cortical neuron layer markers; MAP2: mature neuron marker; TUJ‐1: stained at mature neuronal axon; DCX: newborn neuron marker; PAX6: stained at DF precursor cells; GFAP: labeling astrocytes. C) Staining results of mouse brain sections 4 months after organoid transplantation. Scale bar: 20 µm.

To confirm the cell fate changes in human cells after transplantation, immunostaining analysis was carried out. First of all, fluorescent protein EYFP and human nuclear marker hN were used to identify human cells in the host brain. Cell proliferation marker Ki67 was detected in grafted organoids in 2MPT, which showed there was a progenitor cell pool to provide lineage expansion after grafting. Precursor cells (SOX2^+^) took a considerable part (≈20%) in grafted organoids. In addition, grafted human cells mainly recapitulated dorsal telencephalon identity, which was confirmed by the observation of PAX6. The expression of doublecortin (DCX) showed the generation of a great many newborn neurons (**Figure** **3**B,C). ≈20% of mature human neurons (NEUN^+^ or MAP2^+^) were observed in grafted organoids. Those neurons expressed various cortical layer markers, which suggested the cell fate of the cortex in grafted organoids. Furthermore, human astrocytes took a significant proliferation (4.5% ± 1.7% vs 9.5% ± 0.8%) from 2MPT to 4MPT (Figure S3F,G, Supporting Information), which was consistent with transcriptome analysis.

In summary, through the transcriptome analysis of each sample, human organoids were found to undergo neural lineage development from DO to 2MPT and experience glial lineage proliferation in 4MPT, which was consistent with physiological development process of human fetal brain.

### 2.3 Grafted Human Cells Acquire Properties Similar to the Implanted Host Brain Regions

**Figure 4:**
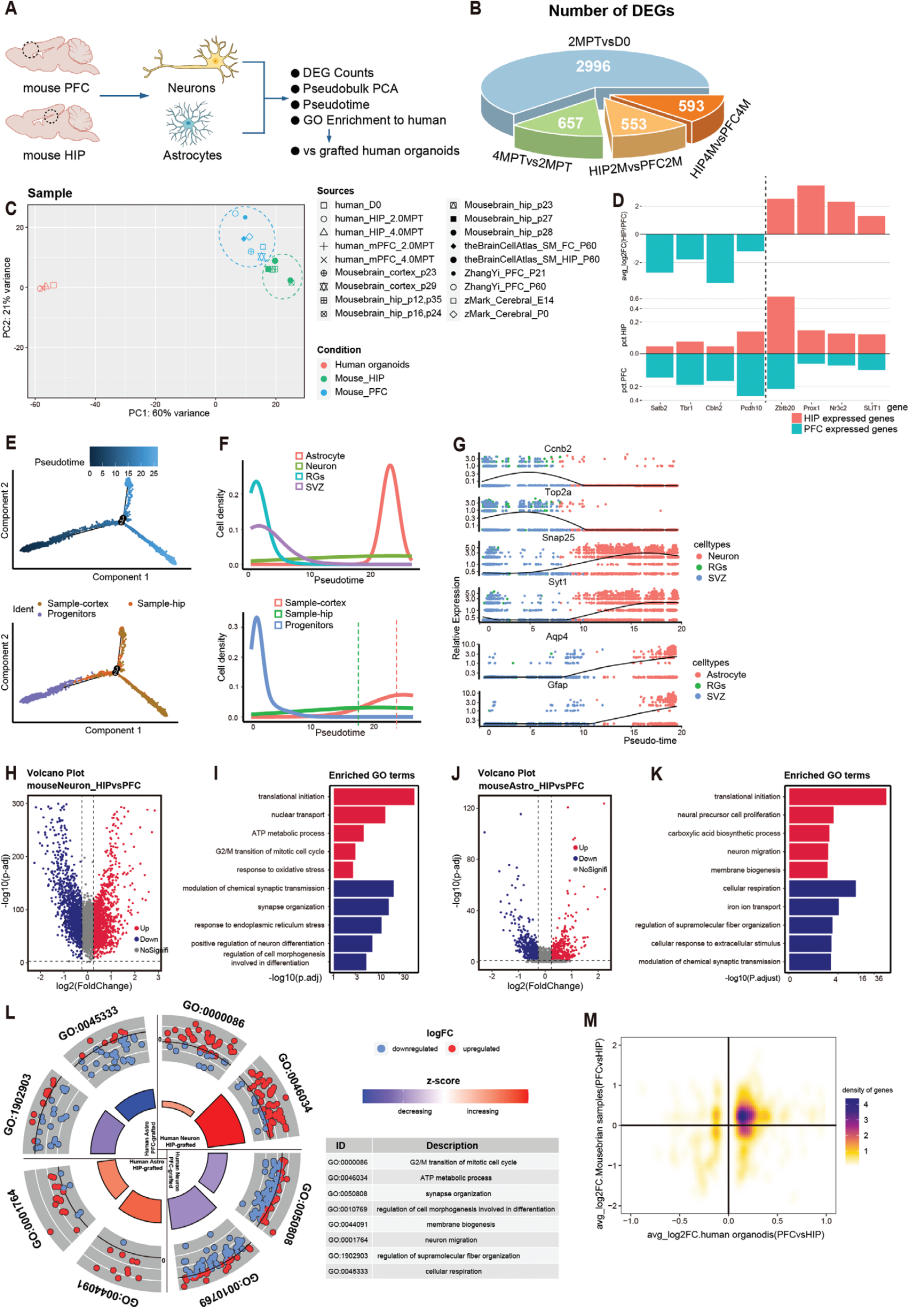
Differences in Human Organoids Grafted into Distinct Brain Regions Resemble Differences in Mouse Brains. A) Schematic diagram showing the difference analysis of the single‐cell samples between PFC and HIP in mice. B) Numbers of DEGs among four groups (P.adjust < 0.05; Percent (PCT)> 0.1; Log_2_FoldChange> 0.25). C) PCA diagram showing the aggregation of pseudo‐bulk transcriptomes between different groups (2 conditions: mouse HIP and mouse PFC) of samples. Left panel: total cells; middle panel: neurons; right panel: astrocytes. p23 represents postnatal day 23. D) Bar chart revealing the expression of HIP or PFC related genes (from public date) in the two representative samples from the Mousebrain Altas database. Log2FC: Log_2_(fold change); PCT: percent. E) Single cell pseudo‐time trajectories of three groups of cells (PFC cells, HIP cells, and Progenitor cells). F) Distribution of pseudo‐time showing that the overall mouse hippocampal cells are less mature than the prefrontal cells. RGs: Radial glial cells; SVZ: Subventricular zone cells. G) Pseudotime analyses for normalized expression of marker genes (RGs, Neurons, and Astrocytes) along the pseudo‐time. H,J) Volcano plot showing the DEGs (HIP vs PFC) in mouse neurons or astrocytes, respectively. Red dots show genes upregulated in HIP; blue dots show genes upregulated in PFC; p‐adj: adjusted *P*‐values. I,K) Representative enriched GO terms (red bar: HIP; blue bar: PFC) of DEGs of mouse neurons and astrocytes, respectively. L) GOCircle plot representing the expression of enriched genes (enriched for GO terms in I or K) of human neurons or astrocytes that grafted into HIP and PFC. The enriched gene sets expressed in humans were consistent with those in mice. M) Density plot of DEGs of neurons showing that DEGs in our and public dataset were predominantly distributed in the first quadrant; x: grafted human organoids in 2MPT (PFC vs HIP); y: mouse brain samples(Mousebrain_cortex_p28 vs Mousebrain_hip_p28, in C).

In order to further investigate the transcriptomic diversity of human cells transplanted in PFC and HIP, these four DEGs were compared with each other (**Figure** **4**B). As the gene number of DEG was generally used to assess the diversity of samples, the number of DEG in different brain region samples (HIP2MPT vs PFC2MPT or HIP4MPT vs PFC4MPT) was almost equiv. to that in time‐series samples (4MPT vs 2MPT), which suggested that the diversity of human cells between brain regions was also necessary to be further analyzed. To characterize DEGs in different brain‐region samples, multiple single‐cell transcriptome datasets of HIP and PFC grafts from adult mice were collected for pseudo‐bulk PCA analysis.^[^ ^17^, ^18^, ^19^, ^20^ ^]^ HIP or PFC samples from adult mice were aggregated respectively, which demonstrated the different cell characteristics at transcriptomic levels (Figure 4C). To simplify the subsequent analysis, two samples (HIP_p28 and PFC_p29 in Figure 4C) from the Mousebrain Atlas were used and their representativeness of the total samples was demonstrated (Figure S4A, Supporting Information). In addition, the identities of these two groups were confirmed by observing the expression of brain region specific genes, such as Satb2, Tbr1, Prox1, and Zbtb20 (Figure 4D).^[^ ^21^, ^22^, ^23^, ^24^, ^25^ ^]^

To determine the distinction between mouse HIP and PFC, neural precursor cells from mouse embryonic telencephalon were analyzed,^[^ ^20^ ^]^ and the PFC matured more during development than the mouse HIP with the comparison of pseudo‐time trajectories (Figure 4E,F), including neurons and astrocytes (Figure S4D, Supporting Information). Additionally, HIP_p28 was also compared with PFC_p23, and PFC_p23 was found to be more mature than HIP_p28 (Figure S4B,C, Supporting Information). Then the DEGs of adult mouse neurons and astrocytes were identified individually (Figure 4H,J), and GO biological process enrichment analysis was performed. GO terms of astrocytes were pointed to three categories of genes: those were crucial for astrocytic development (e.g., HIP: translational initiation; PFC: regulation of fiber organization), astrocyte function (e.g., HIP: carboxylic acid biosynthetic process and membrane biogenesis; PFC: cellular respiration and iron ion transport), interaction of different types of cells (e.g., HIP: neural precursor cell proliferation; PFC: modulation of chemical synaptic transmission) (Figure 4K). In addition, a similar analysis was performed in neurons, and GO terms were also enriched for three clear categories of genes: “neural development”, “neural function”, and “interaction” (Figure 4I). Furthermore, the expression of genes, which presented in the enriched GO terms (all the three categories of GO terms in Figure 4I,K) of the transplanted brain regions, were observed in grafted human cells (neuron or astrocyte), and the transplanted human cells were found to obtain similar transcriptome properties to host brain regions, where the GBOs located (sum of log_2_(fold change) >0 or <0) (Figure 4L), as the genes differentially expressed between grafted human organoids were also largely differentially expressed between PFC and HIP of mouse brain(Figure 4M).

Taken together, transcriptomic divergence in mouse brain regions was identified, and transplanted human‐derived cells were found to acquire properties similar to those of adjacent host brain tissue.

### 2.4 Neurotransmitters Enhance Human Astrocyte Maturation in PFC

**Figure 5:**
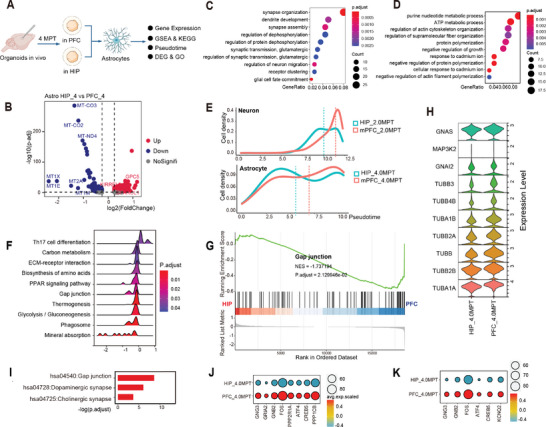
Human Astrocytes are More Mature in PFC_4MPT than in HIP_4MPT due to Neurotransmitter Signaling. A) Differences analysis of grafted human astrocytes between PFC_4MPT and HIP_4MPT. B) Volcano plot showing the DEGs in astrocytes between PFC_4MPT and HIP_4MPT. Red dots represent genes upregulated in HIP_4MPT; blue dots show genes upregulated in PFC_4MPT; the gray dots represent genes that were not significantly differentially expressed between the groups. C,D) The top enriched terms in GO enrichment analysis of upregulated genes in HIP_4MPT (red dots in B) or upregulated genes in PFC_4MPT (blue dots in B) respectively. Color of dots: adjusted *P*‐values; diameter of dots: number of enriched genes. E) Pseudo‐time distribution of human neurons in 2MPT and human astrocytes in 4MPT. Colors show samples; dashed lines show the averages of pseudo‐time distribution. F) GSEA results showing the enriched terms (P.adjust < 0.05, comparison of PFC_4MPT and HIP_4MPT). Colors represent adjusted *P*‐values. G) Differences in the gap junction pathway between PFC_4MPT and HIP_4MPT, indicating a decrease in the HIP_4MPT group. H) Violin plot showing the expression levels of representative genes enriched in the gap junction pathway. I) Human astrocyte enriched top KEGG pathways in PFC_4MPT. Colors represent the ‐log_10_(adjusted *P*). J,K) Expression levels of genes related to dopaminergic (DA) or acetylcholinergic (ACh) synapse pathway between PFC_4MPT and HIP_4MPT, respectively.

Human astrocytes have been implicated various brain disease pathologies.^[^ ^26^, ^27^ ^]^ To determine whether the characteristics of grafted human astrocytes might be affected in different host brain regions, further enrichment analysis was performed. Since the main developmental stage of grafted human astrocytes was 4MPT in our analysis, the DEG among astrocytes between HIP_4MPT and PFC_4MPT (**Figure** **5**B) was acquired. The gene set which was highly expressed in HIP_4MPT was enriched for glial cell fate commitment and neuron maturation (Figure 5C), while the gene set which was highly expressed in PFC_4MPT was enriched for terms related to astrocyte function (Figure 5D), such as ATP metabolic process, response to cadmium ion, cellar response to cadmium ion and regulation of growth. These results suggested that grafted human astrocytes in PFC might be more functionally mature, which was further confirmed by the comparison of pseudo‐time distribution between PFC_4MPT and HIP_4MPT (Figure 5E). Gene set enrichment analysis (GSEA) was also performed, and astrocytes of PFC_4MPT was enriched for Gap junction‐related signaling pathways, which was closely associated with astrocyte function (Figure 5F–H). In addition, neurons in PFC_2MPT were also more mature than those in HIP_2MPT (Figure 5E).

To reveal why transplanted human astrocytes in PFC showed more mature characteristics in transcriptomic level, DEG between PFC_4MPT and HIP_4MPT was obtained and then kyoto encyclopedia of genes and genomes (KEGG) pathway enrichment analysis was performed. The results showed two neurotransmitter synapses (dopaminergic and cholinergic) and gap junctions were in the top enriched KEGG pathways (Figure 5I). Moreover, the high expression of genes in PFC_4MPT was related to dopaminergic and cholinergic synapses (Figure 5J,K), which suggested two neurotransmitters may play important roles in regulating the maturation of grafted human astrocytes.

Together, human astrocytes in PFC_4MPT were found to be more functionally mature at transcriptomic level than HIP_4MPT, which was mainly due to the effects of dopaminergic and cholinergic neurotransmitters.

### 2.5 Morphological Complexity of Both Human and Mouse Astrocytes is Increased in Mouse PFC

**Figure 6:**
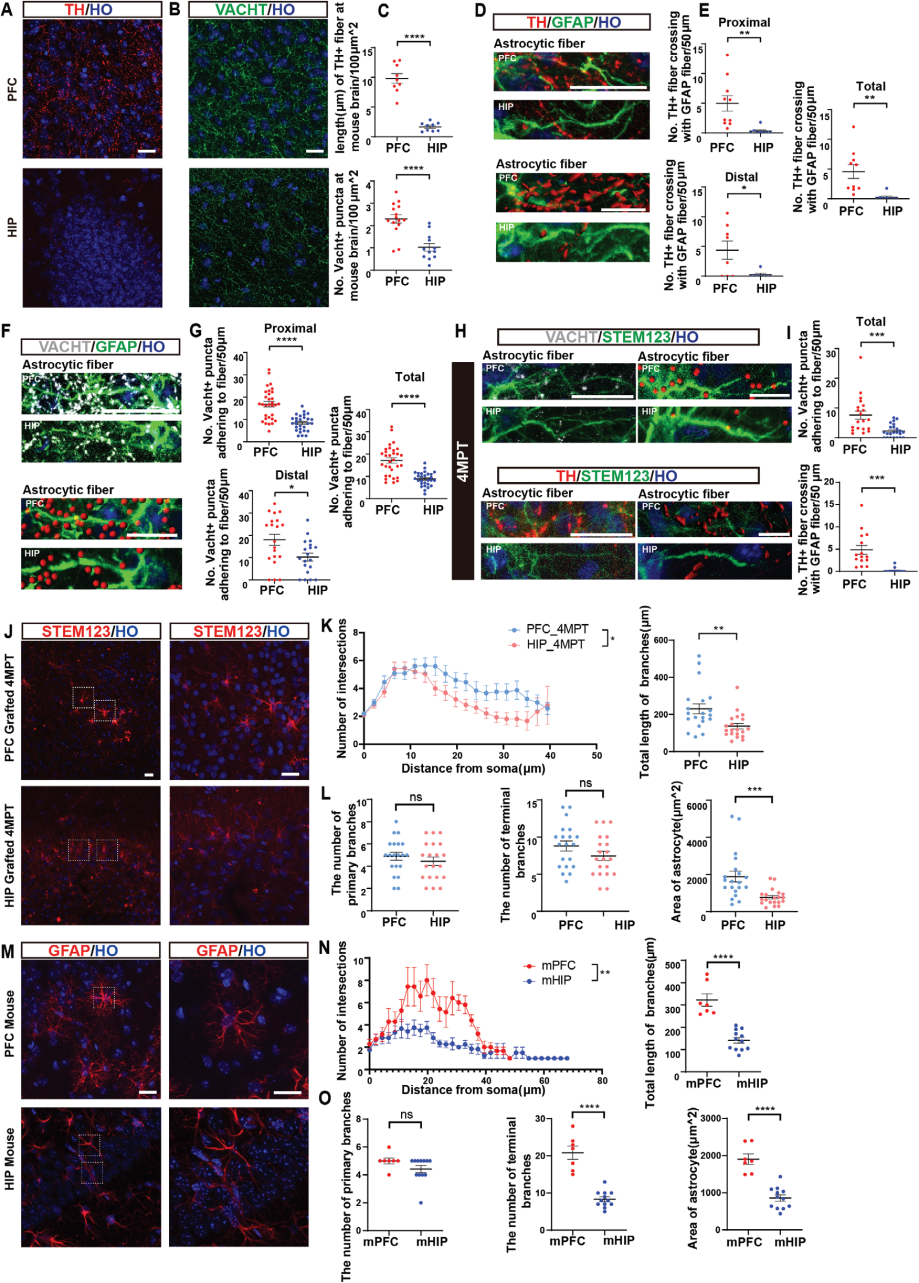
Histological Results Suggest Two Types of Neurons Enhance the Morphological Complexity of Astrocytes after Transplantation. A,B) Immunostaining presenting the density of dopaminergic and cholinergic neurons in PFC and HIP, respectively, in the adult mouse brain. TH: a marker for dopaminergic neurons, VACHT: a marker for cholinergic neurons. C) Comparison of the density of dopaminergic and acetylcholinergic neurons in A or B (*n* = 3 mice). D) Top images: Fluorescence image showing the colocalization of dopaminergic neuron processes and mouse astrocyte fibers in PFC and HIP, respectively. Bottom images: 3D reconstruction images reconstructed by Imaris (not the same astrocyte). GFAP: astrocyte marker. E) Quantification of colocalization of neuron processes and astrocyte fibers (proximal fiber, the distal fiber, and the overall fiber) in Figure D. The boundary (proximal and distal) was 40 µm from the center of the cell body (*n* = 3 mice). F,G) Immunostaining and quantification were performed on acetylcholinergic neurons in PFC and HIP (*n* = 3 mice). H) Representative colocalization images of human astrocyte fibers and mouse neuron processes (acetylcholinergic or dopaminergic neurons) in 4MPT. STEM123: a human astrocyte marker. I) Quantification of colocalization of human astrocyte fibers and mouse neuron processes in H (*n* = 6 mice). J–L) Representative immunostaining images and quantification of the morphological complexity of human astrocyte processes in the HIP or PFC in 4MPT. Right panel in J shows a higher magnification of the boxed astrocytes in left panel in J; Sholl‐analysis, total branch length, number of primary branches, number of terminal branches, and effective area of cell bodies were compared (*n* = 6 mice). M–O) Comparison of morphology of mouse astrocytes in the HIP or PFC was performed, similar to J‐L. mPFC: mouse PFC; mHIP: mouse HIP (*n* = 3 mice). Data are represented as mean ± SEM; scale bar for stained images: 20 µm; scale bars for reconstructed images: 10 µm; two‐way ANOVA in K and N, two‐sided Student's t‐tests in C, E, G, I, L, and O, *p < 0.05, **p < 0.01, ***p < 0.001, ****p < 0.0001.

To analyze the effects of neurotransmitters, DA and Ach, comparison of histology between PFC and HIP of SCID mice was carried out. Dopaminergic neurites (TH^+^) were abundant in the PFC but rarely in the HIP (9.8 ± 0.81 µm/100µm^2^ vs 1.7 ± 0.24 µm/100µm^2^; P < 0.0001). There were more cholinergic positive neurites (VACHT^+^) in PFC than HIP (2.3 ± 0.19/100µm^2^ vs 1.0 ± 0.17/100µm^2^; P < 0.0001) (**Figure** **6**A–C). These results suggested that the higher density of dopaminergic and cholinergic neurites in mouse PFC may contribute to higher expression of these neurotransmitters in the PFC, and hinted that grafted human astrocytes may receive more dopaminergic and cholinergic signals. Adult mouse astrocytes were used to assess the interaction between dopaminergic and cholinergic neurons and astrocytes. Therefore, colocalization of dopaminergic neurites and mouse astrocytes (GFAP^+^) was analyzed. The colocalization was found to be higher in PFC than in HIP on total, proximal (<40 µm from soma), and distal astrocyte fiber (Figure 6D,E), which was consistent with cholinergic neurites (Figure 6F,G; Figure S5C–F, Supporting Information). Meanwhile, human astrocytes (STEM123^+^) were significantly colocalized with both dopaminergic and cholinergic neurons in PFC than in HIP in 4MPT (Figure 6H,I). Therefore, both endogenous host and grafted human astrocytes received more dopaminergic and cholinergic signals in PFC.

Morphological divergence in human astrocytes between PFC and HIP was further analyzed. Morphology of human astrocytes was significantly more complex in PFC than in HIP in 4MPT (*p* = 0.0396). Furthermore, the branch length and effective areas of astrocytes were higher in PFC than in HIP, which also contributed to a more complex morphology (Figure 6J–L). Notably, morphological complexity of human astrocytes did not increase in PFC compared with HIP at 2MPT (*p* = 0.6871) (Figure S5H–J, Supporting Information). Additionally, the mouse astrocyte morphology in PFC and HIP was assessed and the morphological complexity was found to be significantly increased in PFC compared with that in HIP, too (*p* = 0.0057). Moreover, the total branch length, terminal branch number, and effective areas of astrocytes were significantly lower in the HIP than in PFC (Figure 6M–O; Figure S5K, Supporting Information). The above results suggested that grafted human astrocytes in 4MPT showed more complex morphology in PFC than in HIP, similar to the host astrocytes.

In summary, histology results showed the morphology of both human and mouse astrocytes were more complex in PFC than in HIP, which might be due to the higher density of dopaminergic and cholinergic neurons.

### 2.6 Neurotransmitters Enhance Functional Activity and Increase the Morphological Complexity of Astrocytes

**Figure 7:**
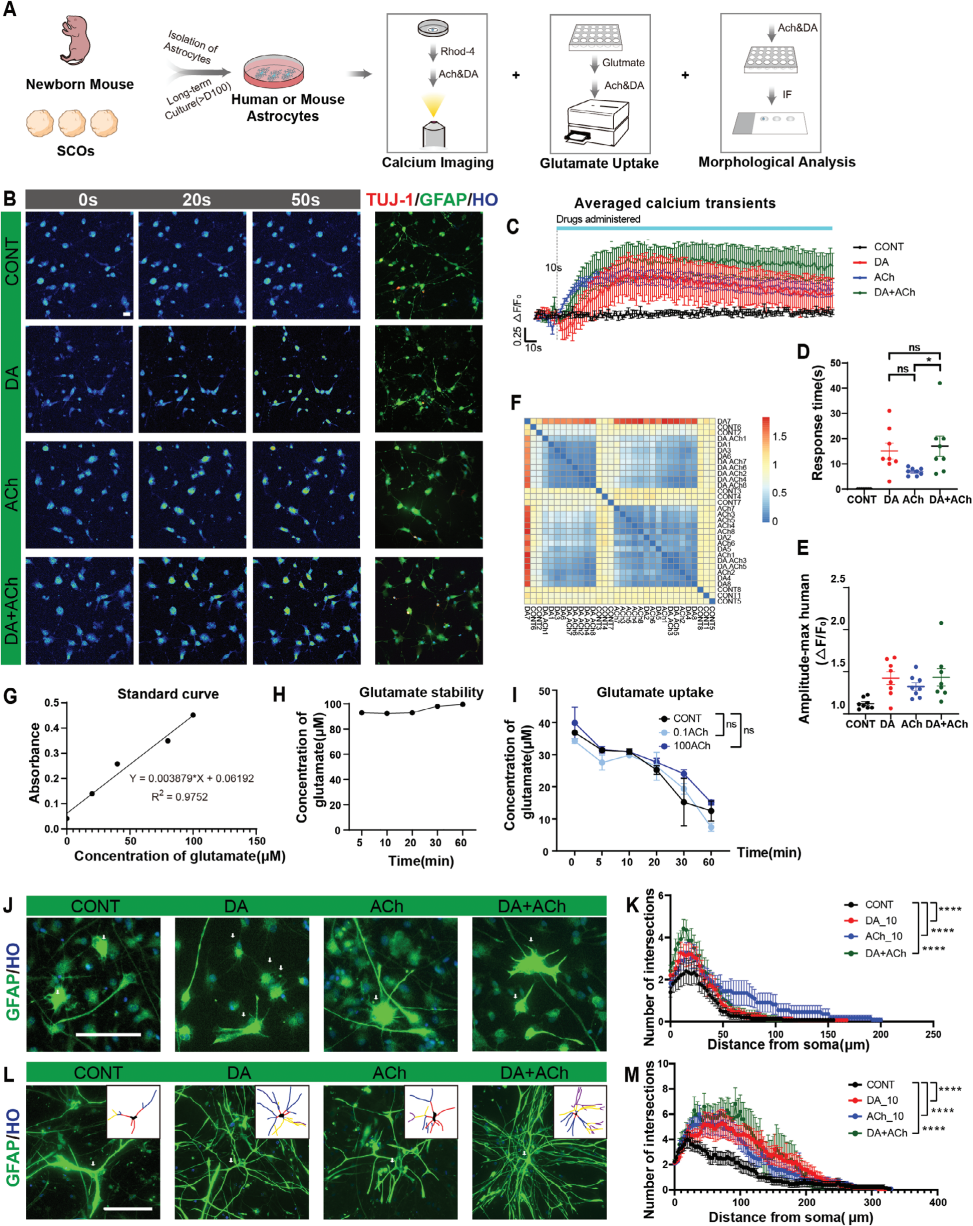
Neurotransmitters Activate Cultured Astrocytes and Increase Their Morphological Complexity. A) Illustration showing the functional tests of human and mouse astrocytes in vitro. B) Representative snapshots of calcium imaging (by Rhod‐4) in human astrocytes at several time points. Left panels: snapshots at 0 s, 20 s, and 50 s in the test are shown respectively. CONT: control group, DA: dopamine group, ACh: acetylcholine group; DA+ACh: both transmitters group. Administration at 10 s. Right panels: Cells in snapshots were verified astrocyte identity by immunostaining. C) Averaged calcium imaging traces. Line represents the mean of fluorescence intensity; colors represent groups (*n* = 8 astrocytes for each group). D,E) Quantification of the response time of calcium signals (confirmed by (F/F0‐1)>10%) or the maximum amplitude of calcium signals in each group (*n* = 8 astrocytes for each group). F) Heatmap showing the correlation of calcium activity among astrocytes in four groups. Samples are clustered unbiasedly; red box indicates high correlation. G–I) Neurotransmitter ACh did not affect glutamate clearance capacity of human astrocytes in vitro (*n* = 3 replicates). J) Representative staining images of human astrocytes in vitro. Neurotransmitters were added for 12 hours. The white arrows indicate representative astrocytes from each group. K) Quantification of sholl‐analysis of four groups in J (*n* = 15 cells for each group). L,M) Comparison of morphology of primary astrocytes from neonatal mice among four groups, similar to J‐K. Insets showing the astrocytes processes tracing of fluorescent images (Cont, *n* = 13; DA, *n* = 14; ACh, *n* = 12; DA+ACh, *n* = 10). Data are represented as mean ± SEM; Scale bars in B: 20 µm; scale bars in J, L: 100 µm; two‐way ANOVA in I, K, and M, two‐sided Student's t‐test in D, *p < 0.05, **p < 0.01, ***p < 0.001, ****p < 0.0001.

To further determine how neurotransmitters (DA and ACh) affect astrocytes astrocyte morphology and functionality, purified human astrocytes were generated from human pluripotent stem cells in vitro.^[^ ^28^ ^]^ Moreover, purified mouse primary astrocytes isolated from neonatal mouse brains were also acquired (**Figure** **7**A; Figure S6F, Supporting Information). Intracellular calcium and glutamate uptake are considered as the major functional indicators of astrocytes.^[^ ^8^, ^29^ ^]^ Therefore, the effect of neurotransmitters on the functional activity of astrocytes was tested. Intracellular calcium was increased dramatically (∆F_DA_/∆F_CONT_ = 3.5; ∆F_ACh_/∆F_CONT_ = 2.7) in human astrocytes (GFAP^+^TUJ‐1^−^) with the treatment of DA or ACh, which indicated neurotransmitters might induce calcium responses and activate human astrocytes (Figure 7B,C,E). Additionally, there was no significant difference in the response time between DA treatment group and Ach treatment group (Figure 7D). Notably, correlation analysis revealed that astrocytes in group ACh and group DA clustered individually, which may indicate that each neurotransmitter has a different effect on astrocytes (Figure 7F). In addition, mouse astrocytes were examined and similar results were observed: both DA and ACh activated mouse astrocytes, no difference in response time, and clustering in correlation analysis (Figure S6A–E, Supporting Information). Furthermore, low concentrations of transmitters (0.1 mM DA or 0.1 mM ACh) did not induce calcium responses in human astrocytes (Figure S6G,H, Supporting Information). So, neurotransmitters may enhance the astrocyte function of intracellular calcium.

Previous studies have shown that mature astrocytes take up neurotransmitters to maintain a homeostasis in synapse, which plays a vital role in preventing excitement toxicity.^[^ ^30^ ^]^ Therefore, a glutamate assay kit was used to assess whether neurotransmitters affected the ability of astrocytes to uptake glutamate in vitro. ACh did not affect the human astrocyte function of glutamate uptake (Figure 7G–I; Figure S7D, Supporting Information).

Furthermore, we examined the morphology of cultured human astrocytes with the treatment of DA or Ach neurotransmitters. Compared with the control group, the astrocytic morphological complexity significantly increased in neurotransmitter groups (Figure 7J,K). The total branch length of human astrocytes also significantly increased(CONT: 112.2 ± 22.4 µm; DA: 175.0±20.8 µm; ACh: 226.5 ± 51.9 µm) (Figure S7A, Supporting Information). Similarly, the morphology of mouse primary astrocytes also significantly increased with the treatment of neurotransmitters (Figure 7L,M). The total branch length and the number of terminal branches increased in the presence of neurotransmitters (Figure S7C, Supporting Information). DA and ACh did not increase the astrocyte stress response, as evidenced by the absence of change in the GFAP^+^ area (Figure S7B, Supporting Information). Furthermore, after the treatment of 0.1 mM ACh, there was no significant change in morphological complexity, the total branch length, or the number of terminal branches of mouse astrocytes (Figure S7E–J, Supporting Information), which may be associated with the results of calcium transient test.

In conclusion, both DA and ACh increased the astrocytic activity, and increased morphological complexity of astrocytes in vitro, which was consistent with the in vivo results after organoid transplantation. The host brain micro‐environment has an effect on the cell fate of similar human organoids, which is mainly due to neurotransmitter signaling.

## 3 Discussion

Recent studies showed that human brain organoids integrated into animal brains after grafting, which provides a new strategy for neural repair. Previously, we reported the survival and neural projection of human brain organoids after transplantation into the mouse cortex, as well as functional integration with host cells and behavioral improvement.^[^ ^7^ ^]^ In this study, we transplanted human brain organoids and obtained the single‐cell atlas of human brain organoids after transplantation, by removing the transcriptomic data from host cells. We did not detect cell overgrowth or pluripotent genes. Above all, we have uncovered a developmental landscape of gene expression and lineage specification, and performed dual decoding of space and time after organoid grafting.

Our research explores the developmental process of human brain organoids after transplantation. Early studies revealed that progenitor cells of the fetal brain first differentiated into neurons and then glial cells.^[^ ^31^, ^32^ ^]^ In this study, we found consistent results in grafted organoids, which indicates that grafted organoids still follow the intrinsic pattern of gene regulation and expression in the host brain. Alternatively, we performed a comparison of correlated brain region samples. The number of DEGs of grafted organoids in HIP and PFC was close to that of grafted organoids at 2MPT and 4MPT. In addition to intrinsic developmental patterns, the host brain environment is important for the cell fate decision of grafted organoids. Previous research showed that the main cell types of HIP and PFC of adult mice are similar;^[^ ^17^ ^]^ thus, we speculate that the brain region variations are related to cell function. Our data verified via enrichment analysis of biological processes as well as pseudo‐time analysis of total cells. Furthermore, we find that transplanted human cells are similar to the host brain region in transcriptional profile.

These results also suggest that there is an unreported repairing mechanism in transplantation therapy. Early studies mainly focused on the exogenous effects of grafted cells to repair CNS, such as nutritional effects, cell renewal, and neuron projection.^[^ ^9^, ^33^, ^34^, ^35^ ^]^ Interestingly, we found transplanted human cells obtained similar transcriptional features to the host brain region, which may be attributed to the effect of the host environment. Given that the fundamental principle of cell transplantation for CNS repair is to regenerate the damaged brain region, the findings suggest that the host environment also plays a crucial role in the endogenous effects of transplantation repair. Furthermore, due to the impact of the environment on transplanted cells, our research results have affirmed the efficacy of the orthotopic transplantation strategy. However, the diseased internal environment may negatively affect cell transplantation.

In this study, we also described the paradigm of fate changes in human brain organoids before and after transplantation. Previous research has indicated there is a substantial alleviation of cell stress response after organoid grafting;^[^ ^16^ ^]^ another report on transcriptional comparison of cortical organoids before and after transplantation showed that the expression of mature neuron genes is upregulated after organoid transplantation.^[^ ^6^ ^]^ In our study, first we made a comparison of samples before and after grafting, time‐series samples, and distinct region samples, and found the transcriptome diversity of the organoids was greatest before and after grafting. Furthermore, we found that this effect may be due to neuron development and maturation, cell projection, and neuron migration as well as an alleviation of hypoxia stress reactions after organoid grafting. Therefore, our research suggests better development and maturation of grafted human cells compared with brain organoids in vitro, which enhances the importance of in vivo animal models for stem cell research.

Our research demonstrated the key role of neurotransmitters DA and ACh in brain region variations. Recently, the brain region variations have been extensively reported, and astrocytes play an important role in it.^[^ ^36^ ^]^ Specifically, astrocytes display diverse morphologies across brain regions.^[^ ^37^ ^]^ In this study, we found the diversity of human astrocytes in PFC and HIP might be due to the endogenous transmitters (DA and Ach), since neither neuron subtype (dopaminergic or cholinergic) was found in our grafted organoids. Then we found distinctions of density of endogenous mouse neurons between brain regions. Moreover, we find the conservativeness of the effect of DA and ACh on both human and mouse astrocytes. Therefore, our results suggest that neurotransmitters are crucial for the brain regions variations in HIP and PFC.

In this study, we also described the vascularization of organoids after transplantation. Successful vascularization was found to be important for organoid survival after grafting.^[^ ^5^ ^]^ We found the blood vessels in grafted human organoids grew from the host, and organoids in 4MPT were more extensively vascularized than 2MPT (Figure S3P,Q, Supporting Information). There was no significant difference in the vascularization of grafted organoids between HIP and PFC groups (Figure S4E–G, Supporting Information).

Our scRNA sequencing data revealed that there was a proportion of neural progenitor cells in organoids after transplantation. However, the majority of progenitor cells in our study were neural precursor cells(high PAX6 and NESTIN expression, low KI67 expression), which are restricted to the fate of neuron lineage, and could serve as a cell pool for regeneration. We found the proportion of cycling cells was ≈2% according to our sequencing data, which may decrease safety concerns. Additionally, there were almost no Ki67‐positive cells among grafted cells, as shown by immunostaining, indicating a low proportion of the devising cells. In addition, we did not observe any malignant proliferation in any of the mice after transplantation.

Our results underscore the effect of neurons on the development and function of astrocytes. Currently, the role of astrocytes in providing nutritional support to neurons has been reported, but the effect of neurons on astrocytes is not yet clear; the effect of neurotransmitters on neurons has been widely studied,^[^ ^39^, ^40^ ^]^ whereas the effects of neurotransmitters on glial cells have been relatively less explored. In our study, two neurotransmitters stimulated the calcium activity of astrocytes and enhanced their morphological complexity. Therefore, we revealed the important role of neurotransmitters in the development of astrocytes, reflecting the complexity of interaction networks between neurons and astrocytes.

In summary, our results report the cell fate changes of brain organoids after transplantation, and revealed that the host micro‐environment might regulate the cell function and fate of astrocytes in grafted organoids. These results might contribute to subsequent in vivo adjustments of cell fate of organoids and regulation of newborn neuron projections, and our study provides a theoretical foundation for the application of human brain organoids in neural repair.

## Conflict of Interest

The authors declare no conflict of interest.

## Author Contributions

Conceptualization was carried out by Y.L. and M.L.; Methodology was prepared by M.L. and S.‐B.X.; Software was dealt with by S.‐B.X., X.L., and Y.H.; Investigation was conducted by S.‐B.X., X.‐R.L., and P.F.; The acquisition of resources was done by Y.C. and X.M.; S.‐B.X., X.L., H.X., S.W., C.C., and L.X.R. helped by Writing – Original Draft; Writing – Review & Editing were done by Y.L., X.G., and M.L.; Visualization was by S.‐B.X. and L.X.R.; Y.L., M.L., and X.G. supervised the project; Y.L. and X.G. performed funding acquisition.

## Supporting information

Supporting Information

Supporting Information

## Data Availability

Raw and processed data of single‐cell RNA‐seq used in this study are available in the Gene Expression Omnibus (GEO) under accession GSE243015.

## References

[1] A. C. Bachoud‐Levi , Prog. Brain Res. 2017, 230, 227. 28552231 10.1016/bs.pbr.2016.12.011

[2] R. H. Baloh , J. P. Johnson , P. Avalos , P. Allred , S. Svendsen , G. Gowing , K. Roxas , A. Wu , B. Donahue , S. Osborne , G. Lawless , B. Shelley , K. Wheeler , C. Prina , D. Fine , T. Kendra‐Romito , H. Stokes , V. Manoukian , A. Muthukumaran , L. Garcia , M. G. Banuelos , M. Godoy , C. Bresee , H. Yu , D. Drazin , L. Ross , R. Naruse , H. Babu , E. A. Macklin , A. Vo , et al., Nat. Med. 2022, 28, 1813. 36064599 10.1038/s41591-022-01956-3 PMC9499868

[3] S. Y. Cao , D. Yang , Z. Q. Huang , Y. H. Lin , H. Y. Wu , L. Chang , C. X. Luo , Y. Xu , Y. Liu , D. Y. Zhu , npj Regen. Med. 2023, 8, 27. 37253754 10.1038/s41536-023-00301-7 PMC10229586

[4] Z. Bao , K. Fang , Z. Miao , C. Li , C. Yang , Q. Yu , C. Zhang , Z. Miao , Y. Liu , J. Ji , Oxid. Med. Cell Longevity 2021, 2021, 6338722. 10.1155/2021/6338722 PMC8629662 34853630

[5] A. A. Mansour , J. T. Gonçalves , C. W. Bloyd , H. Li , S. Fernandes , D. Quang , S. Johnston , S. L. Parylak , X. Jin , F. H. Gage , Nat. Biotechnol. 2018, 36, 432. 29658944 10.1038/nbt.4127 PMC6331203

[6] O. Revah , F. Gore , K. W. Kelley , J. Andersen , N. Sakai , X. Chen , M. Y. Li , F. Birey , X. Yang , N. L. Saw , S. W. Baker , N. D. Amin , S. Kulkarni , R. Mudipalli , B. Cui , S. Nishino , G. A. Grant , J. K. Knowles , M. Shamloo , J. R. Huguenard , K. Deisseroth , S. P. Pasca , Nature 2022, 610, 319. 36224417 10.1038/s41586-022-05277-w PMC9556304

[7] X. Dong , S. B. Xu , X. Chen , M. Tao , X. Y. Tang , K. H. Fang , M. Xu , Y. Pan , Y. Chen , S. He , Y. Liu , Mol. Psychiatry 2021, 26, 2964. 33051604 10.1038/s41380-020-00910-4 PMC8505255

[8] L. C. Faria , F. Gu , I. Parada , B. Barres , Z. D. Luo , D. A. Prince , Neurobiol. Dis. 2017, 102, 70. 28193459 10.1016/j.nbd.2017.01.009

[9] M. Xiong , Y. Tao , Q. Gao , B. Feng , W. Yan , Y. Zhou , T. A. Kotsonis , T. Yuan , Z. You , Z. Wu , J. Xi , A. Haberman , J. Graham , J. Block , W. Zhou , Y. Chen , S. C. Zhang , Cell Stem Cell 2021, 28, 112. 32966778 10.1016/j.stem.2020.08.014 PMC7796915

[10] X. Han , Z. Zhou , L. Fei , H. Sun , R. Wang , Y. Chen , H. Chen , J. Wang , H. Tang , W. Ge , Y. Zhou , F. Ye , M. Jiang , J. Wu , Y. Xiao , X. Jia , T. Zhang , X. Ma , Q. Zhang , X. Bai , S. Lai , C. Yu , L. Zhu , R. Lin , Y. Gao , M. Wang , Y. Wu , J. Zhang , R. Zhan , S. Zhu , et al., Nature 2020, 581, 303. 32214235 10.1038/s41586-020-2157-4

[11] H. J. Kang , Y. I. Kawasawa , F. Cheng , Y. Zhu , X. Xu , M. Li , A. M. Sousa , M. Pletikos , K. A. Meyer , G. Sedmak , T. Guennel , Y. Shin , M. B. Johnson , Z. Krsnik , S. Mayer , S. Fertuzinhos , S. Umlauf , S. N. Lisgo , A. Vortmeyer , D. R. Weinberger , S. Mane , T. M. Hyde , A. Huttner , M. Reimers , J. E. Kleinman , N. Sestan , Nature 2011, 478, 483. 22031440 10.1038/nature10523 PMC3566780

[12] A. Fiorenzano , E. Sozzi , M. Birtele , J. Kajtez , J. Giacomoni , F. Nilsson , A. Bruzelius , Y. Sharma , Y. Zhang , B. Mattsson , J. Emneus , D. R. Ottosson , P. Storm , M. Parmar , Nat. Commun. 2021, 12, 7302. 34911939 10.1038/s41467-021-27464-5 PMC8674361

[13] Y. M. Lee , C. H. Jeong , S. Y. Koo , M. J. Son , H. S. Song , S. K. Bae , J. A. Raleigh , H. Y. Chung , M. A. Yoo , K. W. Kim , Dev. Dyn. 2001, 220, 175. 11169851 10.1002/1097-0177(20010201)220:2<175::AID-DVDY1101>3.0.CO;2-F

[14] S. Zhang , B. S. Zhao , A. Zhou , K. Lin , S. Zheng , Z. Lu , Y. Chen , E. P. Sulman , K. Xie , O. Bogler , S. Majumder , C. He , S. Huang , Cancer Cell 2017, 31, 591. 28344040 10.1016/j.ccell.2017.02.013 PMC5427719

[15] K. A. Lindl , C. Akay , Y. Wang , M. G. White , K. L. Jordan‐Sciutto , Neuropathol. Appl. Neurobiol. 2007, 33, 658. 17931354 10.1111/j.1365-2990.2007.00866.x

[16] A. Bhaduri , M. G. Andrews , W. Mancia Leon , D. Jung , D. Shin , D. Allen , D. Jung , G. Schmunk , M. Haeussler , J. Salma , A. A. Pollen , T. J. Nowakowski , A. R. Kriegstein , Nature 2020, 578, 142. 31996853 10.1038/s41586-020-1962-0 PMC7433012

[17] A. Zeisel , H. Hochgerner , P. Lonnerberg , A. Johnsson , F. Memic , J. van der Zwan , M. Haring , E. Braun , L. E. Borm , G. La Manno , S. Codeluppi , A. Furlan , K. Lee , N. Skene , K. D. Harris , J. Hjerling‐Leffler , E. Arenas , P. Ernfors , U. Marklund , S. Linnarsson , Cell 2018, 174, 999. 30096314 10.1016/j.cell.2018.06.021 PMC6086934

[18] A. Bhattacherjee , M. N. Djekidel , R. Chen , W. Chen , L. M. Tuesta , Y. Zhang , Nat. Commun. 2019, 10, 4169. 31519873 10.1038/s41467-019-12054-3 PMC6744514

[19] A. Saunders , E. Z. Macosko , A. Wysoker , M. Goldman , F. M. Krienen , H. de Rivera , E. Bien , M. Baum , L. Bortolin , S. Wang , A. Goeva , J. Nemesh , N. Kamitaki , S. Brumbaugh , D. Kulp , S. A. McCarroll , Cell 2018, 174, 1015. 30096299 10.1016/j.cell.2018.07.028 PMC6447408

[20] L. Loo , J. M. Simon , L. Xing , E. S. McCoy , J. K. Niehaus , J. Guo , E. S. Anton , M. J. Zylka , Nat. Commun. 2019, 10, 134. 30635555 10.1038/s41467-018-08079-9 PMC6329831

[21] A. Lavado , G. Oliver , Dev. Dyn. 2007, 236, 518. 17117441 10.1002/dvdy.21024

[22] A. Ren , H. Zhang , Z. Xie , X. Ma , W. Ji , D. Z. He , W. Yuan , Y. Q. Ding , X. H. Zhang , W. J. Zhang , J. Physiol. 2012, 590, 4917. 22777671 10.1113/jphysiol.2012.234187 PMC3487045

[23] M. Shibata , K. Pattabiraman , S. K. Muchnik , N. Kaur , Y. M. Morozov , X. Cheng , S. G. Waxman , N. Sestan , Nature 2021, 598, 489. 34599306 10.1038/s41586-021-03952-y PMC9018127

[24] N. Hoshina , E. M. Johnson‐Venkatesh , V. R. Rally , J. Sant , M. Hoshina , M. P. Seiglie , H. Umemori , J. Neurosci. 2022, 42, 4250. 35504727 10.1523/JNEUROSCI.1843-21.2022 PMC9145243

[25] E. Murani , N. Trakooljul , F. Hadlich , S. Ponsuksili , K. Wimmers , Neuroendocrinology 2022, 112, 235. 33853082 10.1159/000516500 PMC8985051

[26] T. Skutella , R. Nitsch , Trends Neurosci. 2001, 24, 107. 11164941 10.1016/s0166-2236(00)01717-3

[27] G. Seifert , K. Schilling , C. Steinhauser , Nat. Rev. Neurosci. 2006, 7, 194. 16495941 10.1038/nrn1870

[28] C. S. Lobsiger , D. W. Cleveland , Nat. Neurosci. 2007, 10, 1355. 17965655 10.1038/nn1988 PMC3110080

[29] R. Krencik , J. P. Weick , Y. Liu , Z. J. Zhang , S. C. Zhang , Nat. Biotechnol. 2011, 29, 528. 21602806 10.1038/nbt.1877 PMC3111840

[30] S. A. Sloan , S. Darmanis , N. Huber , T. A. Khan , F. Birey , C. Caneda , R. Reimer , S. R. Quake , B. A. Barres , S. P. Pasca , Neuron 2017, 95, 779. 28817799 10.1016/j.neuron.2017.07.035 PMC5890820

[31] J. D. Rothstein , M. Dykes‐Hoberg , C. A. Pardo , L. A. Bristol , L. Jin , R. W. Kuncl , Y. Kanai , M. A. Hediger , Y. Wang , J. P. Schielke , D. F. Welty , Neuron 1996, 16, 675. 8785064 10.1016/s0896-6273(00)80086-0

[32] K. Y. Kwan , N. Sestan , E. S. Anton , Development 2012, 139, 1535. 22492350 10.1242/dev.069963 PMC3317962

[33] Y. Tao , S. C. Zhang , Cell Stem Cell 2016, 19, 573. 27814479 10.1016/j.stem.2016.10.015 PMC5127287

[34] S. Pluchino , A. Quattrini , E. Brambilla , A. Gritti , G. Salani , G. Dina , R. Galli , U. Del Carro , S. Amadio , A. Bergami , R. Furlan , G. Comi , A. L. Vescovi , G. Martino , Nature 2003, 422, 688. 12700753 10.1038/nature01552

[35] L. Ma , B. Hu , Y. Liu , S. C. Vermilyea , H. Liu , L. Gao , Y. Sun , X. Zhang , S. C. Zhang , Cell Stem Cell 2012, 10, 455. 22424902 10.1016/j.stem.2012.01.021 PMC3322292

[36] G. H. D. Poplawski , R. Kawaguchi , E. Van Niekerk , P. Lu , N. Mehta , P. Canete , R. Lie , I. Dragatsis , J. M. Meves , B. Zheng , G. Coppola , M. H. Tuszynski , Nature 2020, 581, 77. 32376949 10.1038/s41586-020-2200-5

[37] O. A. Bayraktar , L. C. Fuentealba , A. Alvarez‐Buylla , D. H. Rowitch , Cold Spring Harb. Perspect. Biol. 2014, 7, a020362. 25414368 10.1101/cshperspect.a020362 PMC4292163

[38] D. Lanjakornsiripan , B. J. Pior , D. Kawaguchi , S. Furutachi , T. Tahara , Y. Katsuyama , Y. Suzuki , Y. Fukazawa , Y. Gotoh , Nat. Commun. 2018, 9, 1623. 29691400 10.1038/s41467-018-03940-3 PMC5915416

[39] C. Missale , S. R. Nash , S. W. Robinson , M. Jaber , M. G. Caron , Physiol. Rev. 1998, 78, 189. 9457173 10.1152/physrev.1998.78.1.189

[40] I. Klinkenberg , A. Sambeth , A. Blokland , Behav. Brain Res. 2011, 221, 430. 21108972 10.1016/j.bbr.2010.11.033

