# Supplementary material for: Single‐Cell Transcriptome Landscape and Cell Fate Decoding in Human Brain Organoids after Transplantation
Source: Adv Sci (Weinh). 2024 May 6;11(28):2402287. doi: 10.1002/advs.202402287 (PMC11267311; doi:10.1002/advs.202402287)
Supplement: Supplementary file 1 — Supporting Information [file ADVS-11-2402287-s001.pdf]

ADVANCED SCIENCE
Open Access

Supporting Information

for *Adv. Sci.*, DOI 10.1002/advs.202402287

Single-Cell Transcriptome Landscape and Cell Fate Decoding in Human Brain Organoids after Transplantation

Shi-Bo Xu, Xin-Rui Li, Pan Fan, Xiyang Li, Yuan Hong, Xiao Han, Shanshan Wu, Chu Chu, Yuejun Chen, Min Xu, Mingyan Lin, Xing Guo* and Yan Liu**

Materials and methods

Maintenance of Human Pluripotent Stem Cells *in vitro*

H9: human pluripotent stem cells (ECS: WiCell Agreement NO. 16-W0060) and IMR90-4: human induced pluripotent stem cells (IPS: WiCell Agreement NO. 17-W0063) were plated and cultured in vitronectin (VTN, Thermo Fisher Scientific) coated 6-well plates. E8 medium (Thermo Fisher Scientific), 2-4 ml/well, was half changed every day. Human pluripotent stem cells were cultured for seven days. Clones in good condition presented rounded and smooth edges, and cells inside clones showed a high nucleocytoplasmic ratio. Then cells should be passaged when clones reached about 70-80% confluence. Cells were dissociated with ethylenediaminetetraacetic acid (EDTA, Lonza) at 37 ℃ for 1-2 minutes. EDTA was removed when interstices were observed inside clones under the microscope. Then, clones were gently triturated and added into pre-coated plates. Finally, the plates were shaken gently and then placed back to the incubator. All the cultures were negative for mycoplasma.

Human Brain Organoid Differentiation

Human brain organoids were generated from two pluripotent stem cell lines (H9 and IMR90-4). Organoids obtained for single cell sequencing were induced from the cell line: AAVS-ChR2-EYFP H9 (*1*). Organoids were induced through multiple stages *in vitro*. Specifically, colonies were incubated with dispase (Thermo Fisher Scientific) for 2 minutes at 37 ℃ and gently blown up to keep colonies as intact as possible. On day 0, mixed medium (50% E8 medium mixed with 50% neural induction medium (NIM: DMEM/F12 + 1× NEAA + 1× N2, Thermo Fisher Scientific)) was used to induce the generation of embryoid bodies (EBs). Half-change of NIM was changed every day until the day 7. Then EBs were collected by centrifugation. After the supernatant was removed, fresh NIM containing 10% FBS was added, and then EBs were adhered in 6-well plates and after 8 hours medium was completely removed. From day 10 to day 14, the formation of a rosette-like neural tube structure (ROS) with light and dark distinctions in the middle region of the neuroepithelial tissue could be clearly observed. On day 16, the ROS were gently lifted and then reconstituted in NIM containing 2% B27 (Thermo Fisher Scientific). Brian organoids were generated and subsequently cultured in flasks. On day 20 and day 30, the organoids were sheared with pre-pulled and sterilized Pasteur pipettes. Organoids were cultured until day 42 for sectioning

or transplantation.

Animal Breeding and Organoid Transplantation

The breeding of animals and the operating specifications related to animal experiments followed the guidelines approved by the Laboratory Animal Care and Use Committee and the Laboratory Animal Welfare Ethics Committee of Nanjing Medical University (IACUC-1901051). Mice were kept on a 12-h light/dark cycle, and they had access to food and water ad libitum. Severe combined immunodeficient (SCID) mice which were 6-8 weeks old were randomly divided into different groups. Each group of transplanted mice consisted of 12-15 mice.

Organoids were transplanted following our previously published protocol(*2*). In brief, small (d = 150–250 μm) human brain organoids (derived from cell lines: H9; IMR90-4) were transplanted when cultured to day 42. SCID mice were housed under stable conditions one week prior to transplantation. On the day of grafting, SCID mice were anesthetized with 3% isoflurane in the induction chamber and then fixed on a stereotaxic frame and continuously anesthetized by administration of a low concentration of isoflurane. A minimally invasive craniotomy was performed with a cranial drill. Human brain organoids were carefully aspirated with glass electrodes and subsequently injected into prefrontal cortex (AP = +1.70 mm; ML = ±1.10 mm; DV = −2.7 mm), angled 15° toward the midline, or hippocampus (AP = −2.46 mm; ML = ±2 mm; DV = −2.25 mm) of mice. The human brain organoids transplanted into each site contained approximately 50,000 cells (5 organoids). Mice were then resuscitated on a heating pad until complete recovery. RNA-sequencing or histological analysis was performed in 2 or 4 months after transplantation in mice.

Human Brain Organoid Collection and Sectioning

Brain organoids were washed with DMEM/F12 three times after NIM removal, fixed in fresh 4% paraformaldehyde (PFA, Sigma) for 4 hours and washed with phosphate-buffered saline (PBS, Sigma) three times for 5 minutes. Organoids were submerged in fresh 20% and then 30% sucrose (Sigma) solution (in PBS) overnight. The successfully dehydrated human brain organoids were embedded in optimal cutting temperature (OCT) compound after removing sucrose solution, and sectioned onto glass slides at 10 μm at -20 °C using cryostat. Slides were cryopreserved in a -20 °C storage cabinet for long-term storage.

Organoid Immunofluorescence Staining

Organoid sections were incubated at room temperature and then washed with PBS three times. Organoids were blocked with 1% Triton X-100 (Biolink) and 5% donkey serum (DS, Millipore) for 1 h at room temperature. The sections were incubated overnight in 4 °C refrigerator with primary antibodies prepared in blocking solution . On the next day, organoids were washed with PBS and then incubated with secondary antibodies diluted in 5% DS for 1 h in the dark at room temperature. Organoids were then mounted with Fluoromount-G (Southern Biotech) after being washed three times.

Immunostaining of Mouse Brains

Mouse brains were collected and fixed with fresh 4% PFA at 4 °C overnight. Then brains were dehydrated with 20% and 30% sucrose solutions overnight, successively. The mouse brains were then frozen sectioned at a thickness of 35 μm using a sliding microtome. Brain sections were stored in cryopreservation solution at -20 °C.

Mouse brain sections were placed into 24-well plates. Firstly, the brain sections were blocked with 1% Triton and 5% DS for 1 hour. Then, primary antibody solution was prepared using 0.2% Triton and 5% DS. The next day, brain sections were washed with PBS and then incubated with 5% DS containing secondary antibodies for 1 h, after which sections were kept from light. Lastly, sections were flattened, dried, and mounted onto glass slides.

Single Cell Preparation

Brain sections of the transplanted mice containing the prefrontal cortex or hippocampus, where organoids were grafted, were obtained on ice *ex vivo*. Exogenous tissue with EYFP was dissected and harvested under fluorescence microscope. Additionally, human brain organoids cultured *in vitro* were obtained before transplantation. Then single cell suspensions were obtained. For tissue: incised using tissue scissors and digested with 0.25% Trypsin-EDTA (Life Technology) containing DNase (sigma) for 30 minutes to prepare single cell suspensions. For organoids: digested with TrypLE (Life Technology) for 30 minutes. Cells which passed through cell strainers were reconstituted in DPBS

(Life Technologies) to a final cell concentration of 10^5 for subsequent sequencing (cell viability > 90%).

RNA Library Preparation

Single cell suspensions were coated with oil droplets at single cell level (Chromium Single Cell 3' Chip (10x Genomics, PN-120236)). Gel Beads and Barcodes were used to generate gel beads in emulsions (GEMs). Subsequently, cells were lysed and mRNA was reverse transcribed in individual oil droplets to generate cDNA (S1000 Touch Thermal Cycler (Bio-Rad)). Droplets were then lysed and the cDNA in each oil droplet was individually amplified. cDNA was broken up and cDNA fragment about 200 bp were end-repaired and ligated with adapters, and then screened. Libraries were prepared on a Chromium Single Cell 3' Library & Gel Bead Kit V3 (10x Genomics, 1000075).

Sequencing and Read Acquisition

Sequencing was performed using an Illumina NovaSeq 6000 and raw reads were then obtained with Cell Ranger version 3.1 (10x Genomics). First, FASTQ files corresponding to the samples were generated and then mapped to the reference genome (GRCh38- GRCm39-dual or GRCh38 or GRCm39) for UMI counting. Vacuoles and low-quality sequenced cells were removed. Gene expression matrices for samples were obtained with cell-specific barcode.

Distinguishing Human Cells from Mouse Cells

In order to determinate whether a cell barcode was from human cells or mouse cells, we mapped our data to multiple reference genomes. Specifically, raw reads were aligned to GRCh38-GRCm39-dual genome. For each barcode, the numbers of human and mouse transcripts are indicated. And proportion of human transcripts was presented as h-ratio. According to the distribution of h-ratio in all the cells, 0.2 and 0.8 (h-ratio) were selected to distinguish human and mouse cells. Those cells with ratio between 0.2 and 0.8 were marked as Unknown, and they were speculated as human and mouse mixed cells. Reads were mapped to genome GRCh38 to obtain the transcriptional profile of human-derived cells.

Single Cell Dimensional Reduction and Clustering

Single cells were graphicly clustered through R (R version 4.0.5) package Seurat (version 4.0.4). First, cells with nUMI> 500, nGenes > 500, log10(Genes/UMI) > 0.80, and mitochondrial-related gene expression less than 0.15 were included in clustering analysis. Then, count matrices were normalized using the “NormalizeData” function with LogNormalize method and mitochondrial content across cells were moved using the SCTransformed function. Samples were integrated by the functions “Findneighbors” and “IntegrateData” on 2,500 highly variable genes (reduction: rpca). Then top 40 principal components (PCs) were chosen to visualize cell distribution in the uniform manifold approximation and projection (UMAP) plot. Single cells were clustered by the function “FindClusters”. Finally, the identity of each cell cluster was distinguished by conserved markers. Generally, graphs were generated using the R package ggplot2 (version 3.3.5).

Cell Type Notation

In order to determine the cell identity of each cell cluster, we mapped our cells to the human tissue single cell transcriptome database (SCHCL)(*3*). First, all the human cells were hierarchically clustered through serial resolutions. Then, we mapped clusters at each resolution to the public database, and three types of human cells owning the highest score were included in the heat plot to predict cell identities. Lastly, both conserved markers of each cell cluster and characteristic genes expressed during neural differentiation were observed to confirm the expression of these major cell lineages before and after the transplantation of human brain organoids.

Constructing Single Cell Trajectories

The single cell pseudo-time trajectories of all the human or mouse cells were reconstructed using R package Monocle3 (version 1.0.0) for total cells or Monocle2 (version 2.18.0) for subtypes . The Seurat object was transformed to Monocle dataset, and genes expressed less in 10 cells were screened and removed. The top 1,000 genes expressed most significantly differentially were extracted to order cells. All the cells were dimensionally reduced and then mapped to plots by the function "DDRTREE" or "UMAP" and were ordered in the trajectories by the function "Ordercells" or "Order_cells". The differentially expressed genes along the pseudotime trajectory were performed by the function “differentialGeneTest”. Typical genes were chosen to confirm the lineage specification.

Pseudo-time trajectories of human cells were also obtained using R package Slingshot (version 1.8.0).Firstly, dimension-reduced data of the Seurat object was mapped. Then the minimum spanning tree was constructed. According to the trajectory distances between the starting point and the cells, all the cells were allocated a Pseudo-time and projected on the curve by the function "Slingshot". We verified pseudo-time trajectories obtained by multiple methods were generally consistent.

Mapping to mouse brain regions

Each sample of single cell transcriptome data before and after transplantation was unbiasedly mapped to E13.5 mouse brain from Allen Brain Atlas database using the Voxhunt packet (version 1.0.0). Firstly, the top 100 characteristic genes of each brain region were obtained, then transcriptome similarities of sample to the brain region were calculated by the function "Voxel_map", and samples were mapped to the sagittal and coronary sections of brain (Pearson's correlations). Simultaneously, the correlation coefficients between each sample and each brain region of E13.5 mouse were calculated and visualized through heat map.

Single Cell Transcriptome Comparison of Organoids and Human Fetal Tissue

Transcriptome datasets used in this study were compared with public time-series datasets of single cell human fetal tissue from Brainspan (*4*). After raw counts matrices were normalized, the average expression of all genes of each sample was calculated. Furthermore, each sample was compared with human fetal tissue from the Brainspan database to perform correlation analysis (Pearson's correlations). Visual analysis was performed using Pheatmap package (version 1.0.12).

Correlation Analysis among Organoid Samples

Principle Composition Analysis (PCA): after normalization, gene expression matrices were accumulated to obtain the average expression of single cell of each sample. We used the DESeq2 R package (version 1.30.1) to perform pseudo-bulk analysis. In short, DESeq2 objects were constructed by the function "DESeqDataSetFromMatrix". Genes expressed less in 10 cells were excluded from analysis. Then differential expression test and variance stabilizing transformation

were performed by the function "DESeq", and PCA plots were generated with the function "PlotPCA".

Similarity Analysis: a distance matrix, which presented the transcriptional similarity among organoids samples, of all the samples was obtained by the function "Dist" on the DESeq2 object. The distance matrix was displayed by the function " Pheatmap". Samples were unbiasedly clustered by distance.

Identification of Differentially Expressed Genes

To obtain differentially expressed genes (DEGs) between samples or cell types, we used the function "FindMarkers" (Method = MAST (version 1.18.0)), which is suitable for single cell transcriptional data. Then, genes with a percentage >0.1, Log2foldchange >0.25, and adjusted P-values <0.05 were chosen for follow-up differences analysis. Volcano plots were produced by the function “Geom_point” using R package Ggplot2.

Enrichment Analysis

After the DEG list was obtained, enrichment analysis was performed using the R package ClusterProfiler (version 4.1.4)(5). Such as: Gene Ontology analysis (GO) and the signaling pathway enrichment analysis, KEGG, were also performed. Human reference genome was used for mapping, and the point diagrams or bar charts were obtained by functions “Barplot” or “Dotplot”, respectively. Additionally, description terms and P values were extracted to create a new plot.

GO enrichment analysis comparison of human samples and mouse brain tissue was performed by the function “CompareCluster”. Then enriched terms were summarized and classified into three categories (cell fate determination, cell function, and cell subtype interaction). Genes enriched in each representative terms of mouse HIP or PFC were extracted, and then they were observed in human samples. Results were visualized by the function " GOCircle" using GOplot.

Gene Set Enrichment Analysis

Firstly, cell subtypes were extracted (such as astrocytes), and a list of DEGs (HIP_4MPT vs PFC_4MPT) was then created. Then Genes were sorted by Log2foldchange in the list. The sorting

gene list was used to perform the gene set enrichment analysis (GSEA) by the function “GseKEGG”. The results of enrichment analysis were visualized by the function "RidgePlot".

Primary Astrocytes Acquisition and Astrocytes Differentiation

Cortical tissues were separated from the newborn mice (C57BL/6). Then tissue was mechanically crushed and filtered through cell strainers to obtain primary single cells. The primary cells were cultivated in a T75 flask (Thermo Fisher Scientific) 7-8 days later, and then was gently shaken at 37 ℃ overnight to remove the neurons, oligodendrocytes and microglial cells. The remaining was re-suspended and cultivated to obtain pure astrocytes. Mouse astrocytes were cultured in NIM containing 10 ng/ml EGF, 10 ng/ml bFGF and 1× penicillin-streptomycin solution (PS, Life technology). We subsequently verified the astrocyte identity by immunofluorescence and calculated the percentages of the total astrocytes.

The acquisition of human-derived astrocytes referred to the previously established method(*6*). Human stem cells were used to differentiate high-purity astrocytes *in vitro*. EGF (10 ng/ml, Life technology) and bFGF (10 ng/ml, Life technology) were added to the cultivation of astrocytes.

Calcium Activity Test and Analysis

In order to simulate the vesicle release of neurotransmitters in synaptic cleft, neurotransmitters at a concentration of mM were used to test the calcium response of the astrocytes (*7, 8*). Human or mouse astrocytes were cultivated on the confocal dishes *in vitro* for 7 days. Then dishes were divided into four groups: Control group (PBS), DA group, ACh group, DA+ACh group. On the testing day, astrocytes were incubated in a Rodh-4 calcium ion fluorescent indicator solution at 37 ℃ for 30 minutes. Severe vibrations that may have disturbed the test were avoided. Then astrocytes on the confocal dishes were transferred to the recording room, and calcium activity was recorded under the LSM800 (ZEISS). The entire test lasted for 150s. At the 10s, 100mM transmitters (DA, ACH) or PBS was added into dish. The low concentration group meant 0.1mM. After recording, ImageJ were used to measure and analyze fluorescent signals. Single astrocytes were manually selected to measure the changes in fluorescent intensity. F0 was defined as the average fluorescent intensity per second under the baseline fluorescent conditions.

Glutamate Clearance Assay

Human astrocytes were adhered on a 24-well plate with a cell density of 30,000 cells per well. The stability of glutamate was confirmed by detecting the concentration changes of glutamate solution in the blank well without astrocytes under experimental conditions. A glutamate Assay Kit (Sigma, Mak004) was used to detect the concentration of glutamate solution. Astrocytes were divided into three groups: CONT group, 100ACh group, and 0.1ACh group. Before the assay, Astrocytes were equilibrated with HBSS Buffer (Thermo Fisher Scientific) for 30 minutes. At the beginning of test, glutamate solution (about 40 uM, in HBSS) was added into each group simultaneously. Then concentration of glutamate was measured in one hour. As neurotransmitters DA produced soluble black substances during the test which disturbed the detection of glutamate, group DA was excluded.

Morphology Analysis after Adding Neurotransmitters

Human or mouse astrocytes were cultured *in vitro* for one week, then DA or ACh (10 mM or 0.1 mM) was added into cultures for 12 hours. The cells were then fixed in fresh 4% PFA for immunofluorescent staining. Astrocytes was marked by the antibody GFAP. The semi-automatic Sholl Analysis was used to analyze the morphological complexity of astrocytes in each group (*9*). In short: ImageJ was used to manually extract the cell body and process of single astrocytes; reconstruct the flat distribution of astrocyte process; label the nucleus center as the center of circles with a radius of 2.5*n μm (n = 1,2,3 ...); calculate the number of intersections of astrocyte processes and each circle, and construct a distribution curve for comparison.

Statistical Analysis

In order to calculate the proportion of specific molecules in human-derived cells in the transplanted mouse brain slices, at least 3 brain slices from at least 3 transplanted mice were used. The total cell number was confirmed by the human nuclear marker hN. The experiments of transplantation were repeated using two stem cell lines (IMR90-4 and H9).

Data are presented as mean ± SEM. The statistical analysis was performed using software Prism 8 (GraphPad). Significance was determined using the Student's t-test or two-way ANOVA, $p<0.05$ was marked as *, $p<0.01$ was marked as **, $p<0.001$ was marked as ***, and $p<0.0001$ was marked as ****.

Data availability

Raw and processed data of single-cell RNA-seq used in this study are available in the Gene Expression Omnibus (GEO) under accession GSE243015.

Ref:

1. M. Xiong, Y. Tao, Q. Gao, B. Feng, W. Yan, Y. Zhou, T. A. Kotsonis, T. Yuan, Z. You, Z. Wu, J. Xi, A. Haberman, J. Graham, J. Block, W. Zhou, Y. Chen, S. C. Zhang, Human Stem Cell-Derived Neurons Repair Circuits and Restore Neural Function. Cell stem cell 28, 112-126 e116 (2021).
2. X. Dong, S. B. Xu, X. Chen, M. Tao, X. Y. Tang, K. H. Fang, M. Xu, Y. Pan, Y. Chen, S. He, Y. Liu, Human cerebral organoids establish subcortical projections in the mouse brain after transplantation. Mol Psychiatry 26, 2964-2976 (2021).
3. X. Han, Z. Zhou, L. Fei, H. Sun, R. Wang, Y. Chen, H. Chen, J. Wang, H. Tang, W. Ge, Y. Zhou, F. Ye, M. Jiang, J. Wu, Y. Xiao, X. Jia, T. Zhang, X. Ma, Q. Zhang, X. Bai, S. Lai, C. Yu, L. Zhu, R. Lin, Y. Gao, M. Wang, Y. Wu, J. Zhang, R. Zhan, S. Zhu, H. Hu, C. Wang, M. Chen, H. Huang, T. Liang, J. Chen, W. Wang, D. Zhang, G. Guo, Construction of a human cell landscape at single-cell level. Nature 581, 303-309 (2020).
4. H. J. Kang, Y. I. Kawasawa, F. Cheng, Y. Zhu, X. Xu, M. Li, A. M. Sousa, M. Pletikos, K. A. Meyer, G. Sedmak, T. Guennel, Y. Shin, M. B. Johnson, Z. Krsnik, S. Mayer, S. Fertuzinhos, S. Umlauf, S. N. Lisgo, A. Vortmeyer, D. R. Weinberger, S. Mane, T. M. Hyde, A. Huttner, M. Reimers, J. E. Kleinman, N. Sestan, Spatio-temporal transcriptome of the human brain. Nature 478, 483-489 (2011).
5. T. Wu, E. Hu, S. Xu, M. Chen, P. Guo, Z. Dai, T. Feng, L. Zhou, W. Tang, L. Zhan, X. Fu, S. Liu, X. Bo, G. Yu, clusterProfiler 4.0: A universal enrichment tool for interpreting omics data. Innovation (Camb) 2, 100141 (2021).
6. R. Krencik, J. P. Weick, Y. Liu, Z. J. Zhang, S. C. Zhang, Specification of transplantable astroglial subtypes from human pluripotent stem cells. Nat Biotechnol 29, 528-534 (2011).
7. J. Kleinle, K. Vogt, H. R. Luscher, L. Muller, W. Senn, K. Wyler, J. Streit, Transmitter concentration profiles in the synaptic cleft: an analytical model of release and diffusion. Biophys J 71, 2413-2426 (1996).
8. J. W. Mozrzymas, E. D. Zarnowska, M. Pytel, K. Mercik, Modulation of GABA(A) receptors by hydrogen ions reveals synaptic GABA transient and a crucial role of the desensitization process. J Neurosci 23, 7981-7992 (2003).
9. K. E. Binley, W. S. Ng, J. R. Tribble, B. Song, J. E. Morgan, Sholl analysis: a quantitative comparison of semi-automated methods. J Neurosci Methods 225, 65-70 (2014).

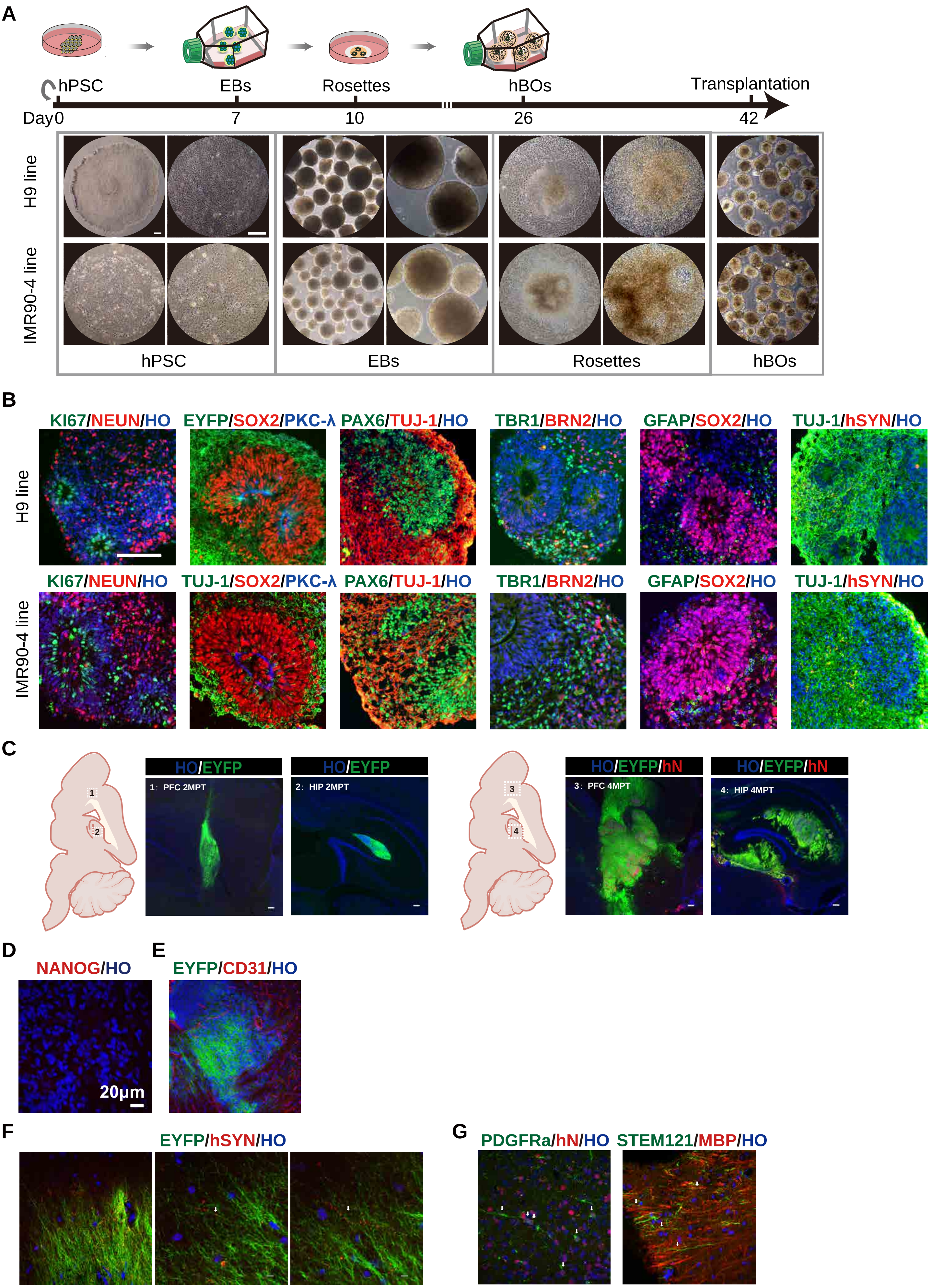

Supplementary Figure 1

Figure S1 Acquisition of hBOs for Transplantation *in vitro*

(A) Schematic diagram of *in vitro* differentiated hBOs and bright-field images in DIC microscopy. The diagram showing the four stages of differentiation (hPSC, EBs, Rosettes, hBOs).

(B) Immunostaining demonstrating that molecular features of hBOs. Mature brain organoids were characterized by anatomical laminations similar to the developing human brain. Ventricular zone (VZ) and subventricular zone (SVZ) were stained for KI67 (a proliferating cell marker) and SOX2 (a progenitor cells labeling), and PKC-λ was stained in the apical surface of VZ. Mature neuron maker TUJ-1 and NEUN showed the cortical plate (CP) in hBOs. A number of $PAX6^+$ cells were detected which suggested the identified cell fate of telencephalon. As well, hBOs expressed cortical neural markers, which were similar to human brain cortex. These markers include TBR1(a deep-layer neuron marker) and BRN2(a layer III neuron marker).

(C) Immunostaining showing the location of the grafts (in Figure 1B).

(D) Little NANOG expression (a pluripotent marker) was detected in human-derived cells (identified by EYFP) after transplantation.

(E-G) hBOs survived and integrated in the host brain after transplantation.

Scale bar in A, B, C: 100 μm; Scale bar in D, E, F, G: 20 μm;

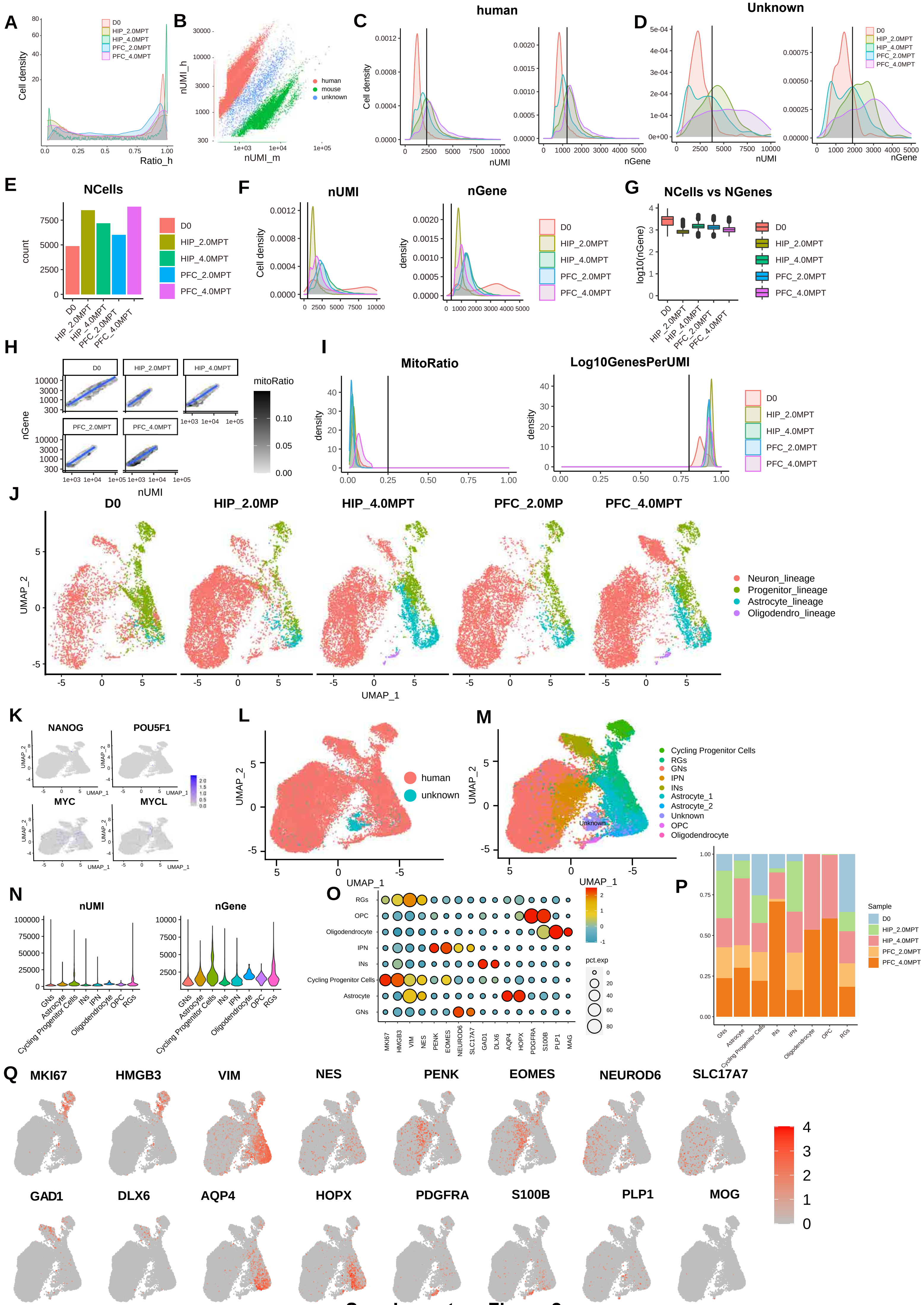

Supplementary Figure 2

Figure S2 Quality Criteria and Detailed Subtypes of Human-derived Cells

(A) Density distributions showing the proportion of human transcripts of each sample, which obtained from the human-mouse dual reference genome. X-axis represents the proportion of human transcripts in a single cell; y-axis represents the density of cells; colors represent samples.

(B) Scatter plot showing distribution of human and murine transcripts in all cells. Colors represent groups (divided according to Ratio-h value (0.2 and 0.8)).

(C and D) Density distribution of the number of expressed genes or transcripts in Human Cells or Unknown Cells (as in B). The black lines in C: represent averages of 2,262 and 1,232; in D: represent averages of 3,764 and 1,883.

(E-I) Samples showed similar and good quality after screening out low-quality cells.

(J) UMAP plot divided by groups. Colors represent cell types.

(K) Human cells express little pluripotency-related genes after transplantation.

(L) Unknown Cells are clustered together and separated from Human Cells after global clustering.

(M) UMAPplot showing subtype cell identification. Cycling Progenitor Cells (CPCs), Radial glial cells (RGs), Intermediate progenitor neuron (IPN), Glutamatergic neural cells (GNs), Inhibitory neural cells (INs), Oligodendrocyte progenitor cells (OPCs) and Oligodendrocytes (Oligos).

(N-Q) Expression profiles of various subtypes after detailed cell identification, including Cycling Progenitor Cells: MKI67, HMGB3; Radial glial cells: NIM, NES; Intermediate progenitor neuron: PENK, EOMES; Glutamatergic neural cells: NEUROD6, SLC17A7; Inhibitory neural cells: GAD1, DLX6; Oligodendrocyte progenitor cells: PDGFRA, S100β; Oligodendrocytes: PLP1, MAG.

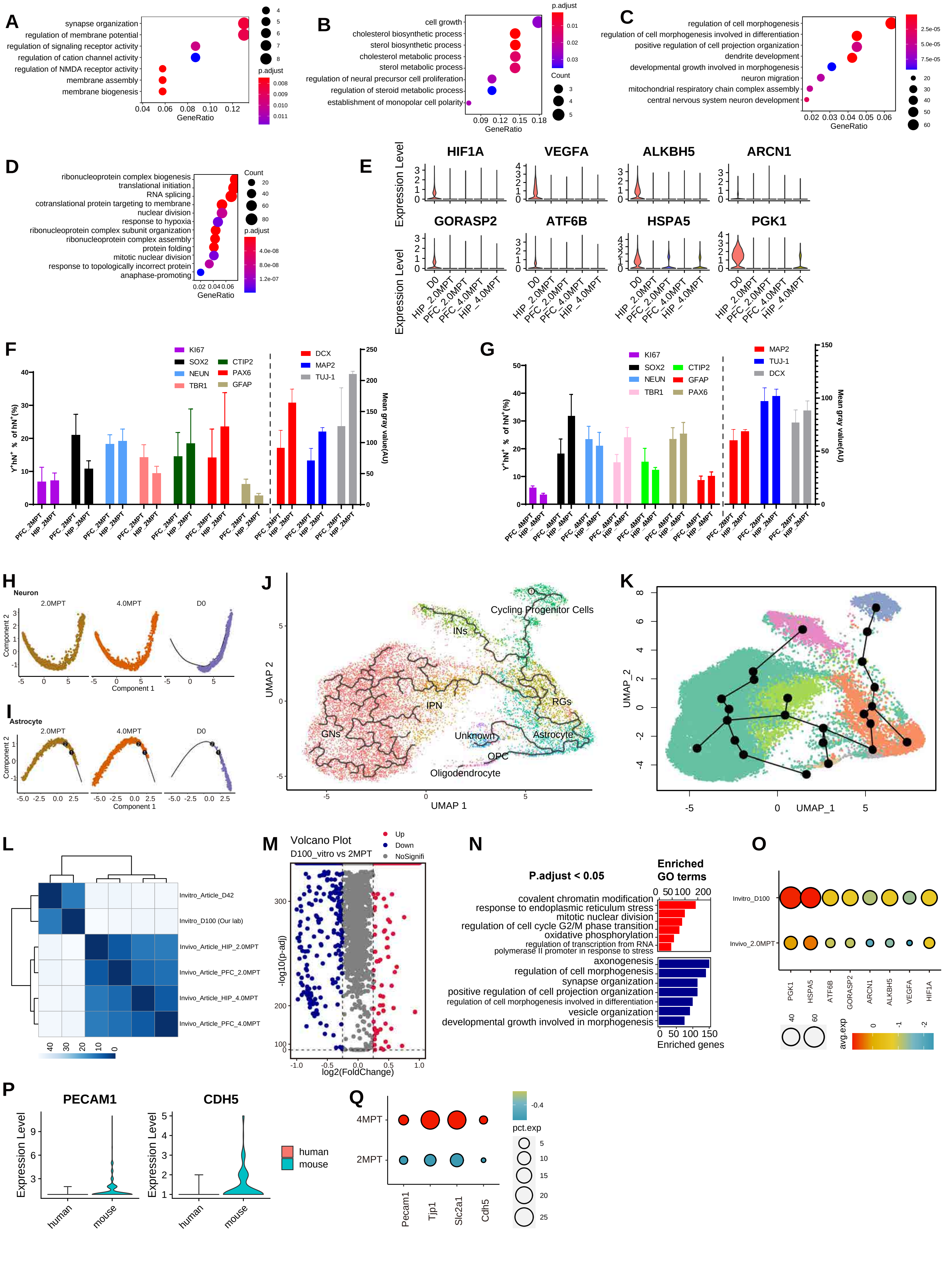

Supplementary Figure 3

Figure S3 Difference Analysis between Samples in Each Group

(A) GO enrichment results of the gene set in the first quadrant in Figure 2E.

(B) Top GO terms enriched by the gene set in the third quadrant in Figure 2E.

(C) GO enrichment results for a gene set expressed only in the 2MPT group in Figure 2E.

(D) GO enrichment results for a gene set expressed only in the D0 group in Figure 2E.

(E) Violin plot showing the expression of cellular stress-related genes in each sample.

(F and G) Quantification of staining results in Figure 3B and 3C, respectively. (*n* = 36 mice transplanted with hBOs, *n* > 72,000 cells).

(H and I) Pseudo-time trajectories divided by groups in Figure 3G and 3J individually.

(J and K) Single-cell trajectory analysis of all the human cells. Using R packages Monocle 3 and Slingshot respectively.

(L) Heat map showing human brain organoids *in vitro* were separated from organoids *in vivo*; ScRNA-seq sample cultured *in vitro* for about 100 days was added: Invitro_D100 (OurLab).

(M) Volcano plot showing the differentially expressed genes (sample D100 *in vitro* vs samples 2MPT *in vitro*).

(N) GO term analysis (red bar: red dots in M) showing that transplanted organoids were mainly enriched for neuron growth and maturation, stress response alleviation.

(O) Dot plot revealing stress-related gene expression in samples.

(P) Violin plot showing blood vessels in grafted organoids composed of mouse cells.

(Q) Dot plot revealing that 4MPT organoids were more extensively vascularized than 2MPT; endothelial cell markers: Pecam1(Cd31), Cdh5(Cd144), Tjp1(Zo-1), and Slc2a1(Glut1).

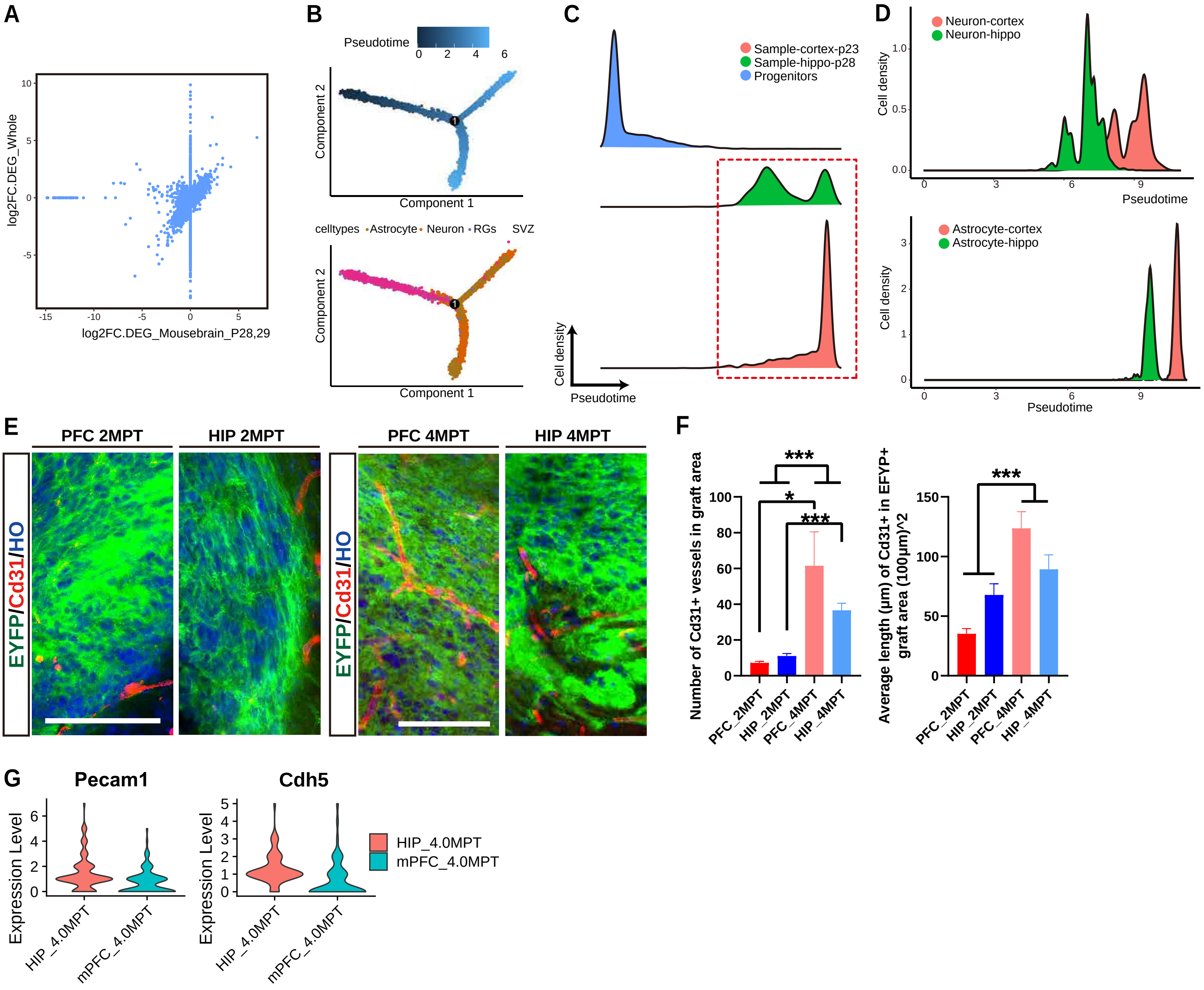

Supplementary Figure 4

Figure S4 Mouse PFC are More Functionally Mature than HIP

(A) Scatter plot revealing the linear correlation of gene expression between representative samples (x-axis: Mousebrain_HIP_P28 and Mousebrain_PFC_P29 in Figure 4C) and two groups (y-axis: overall mouse HIP and PFC samples in Figure 4C).

(B) Pseudo-time trajectories of samples (Progenitors, HIP_P28, and PFC_P23) (by Monocle2).

(C) Pseudo-time distribution of samples (same as B).

(D) The pseudo-time distribution of neurons (top panel) and astrocytes (bottom panel) in sample HIP_P28 and sample PFC_P29.

(E and F) Representative immunofluorescence images and quantification of vascularization of human organoids. EYFP was stained in human cells; Cd31 is a marker for vascular endothelial cells. (n = 8 mice transplanted with hBOs, n > 90,000 human cells).

(G) Violin plot showing there was no significant difference in vascularization between organoids 4MPT grafted in HIP and PFC.

Scale bar in E: 100 μm.

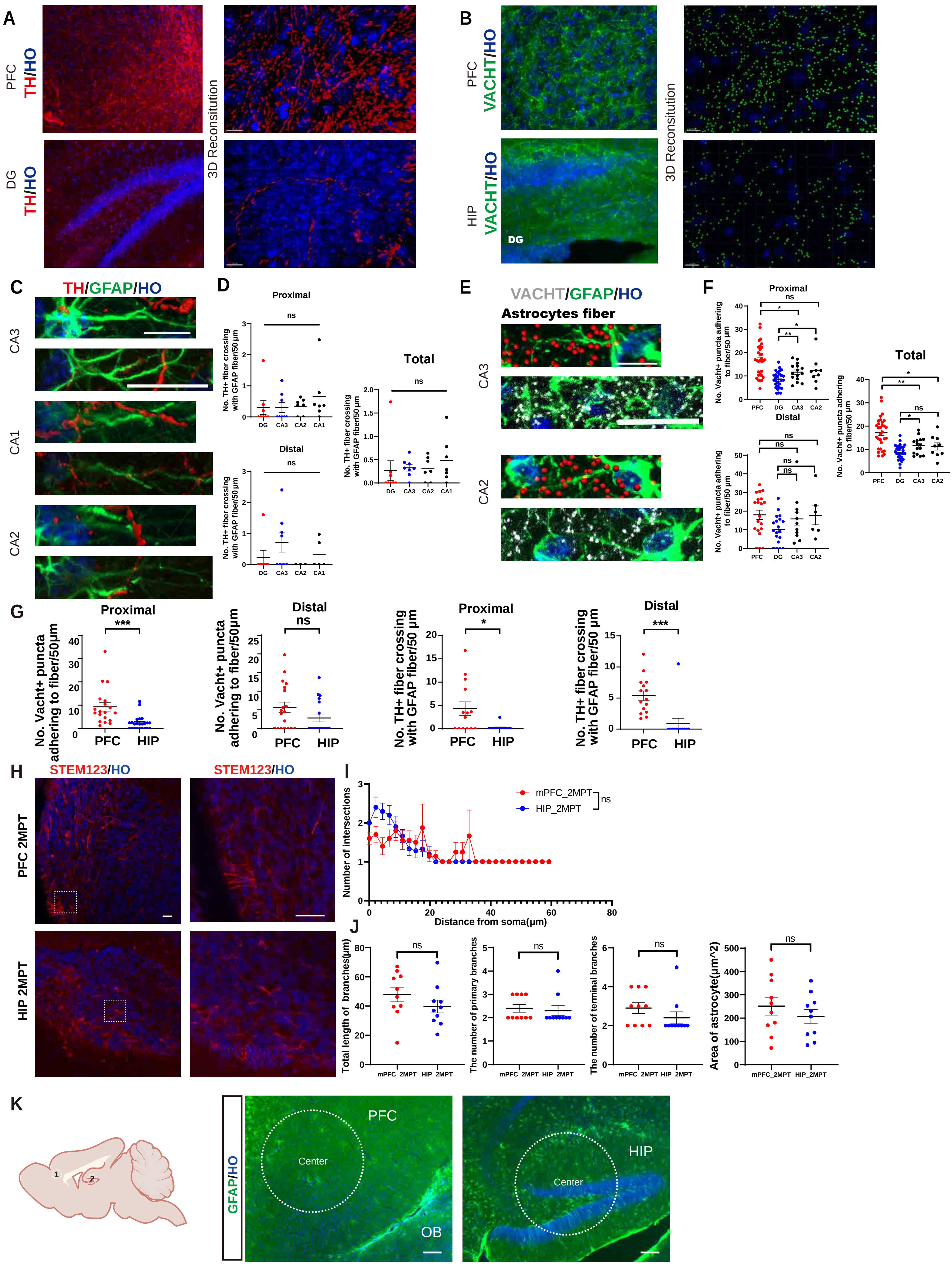

Supplementary Figure 5

Figure S5 Histological Comparison of Neurotransmitters between PFC and HIP

(A and B) Representative staining images and 3D reconstruction of dopaminergic neurons (TH^{+}) and acetylcholinergic neurons (ACh^{+}) in PFC and HIP.

(C and E) Representative images of colocalization of neuron processes (dopaminergic and acetylcholinergic neurons) and mouse astrocytes within the sub-regions of HIP.

(D and F) Quantification of colocalization (in C and E) on proximal, distal, and total astrocyte fibers. The colocalization of GFAP and DA kept low in each sub-region in HIP; the colocalization of GFAP and VACHT on total fibers was higher in PFC than in each sub-region in HIP (DG, CA2, CA3).

(G) Quantitative results corresponding to Figure 6H ($n = 6$ mice).

(H-J) Representative immunofluorescence images and quantification of morphology of human astrocytes in 2MPT, Similar to Figure 6J-L ($n = 6$ mice).

(K) Staining images showing the locations of the mouse or human astrocytes (in Figure 6M and 6J) selected for quantification. These astrocytes are located near the injection site (about 300 μm); OB: olfactory bulb.

Data are represented as mean ± SEM; scale bar for staining images in A-H: 20 μm, in K: 100 μm; scale bar for reconstruction images: 10 μm; two-way ANOVA in I, two-sided Student's t-test in D, F, G, and J, *$p < 0.05$, **$p < 0.01$, ***$p < 0.001$, ****$p < 0.0001$.

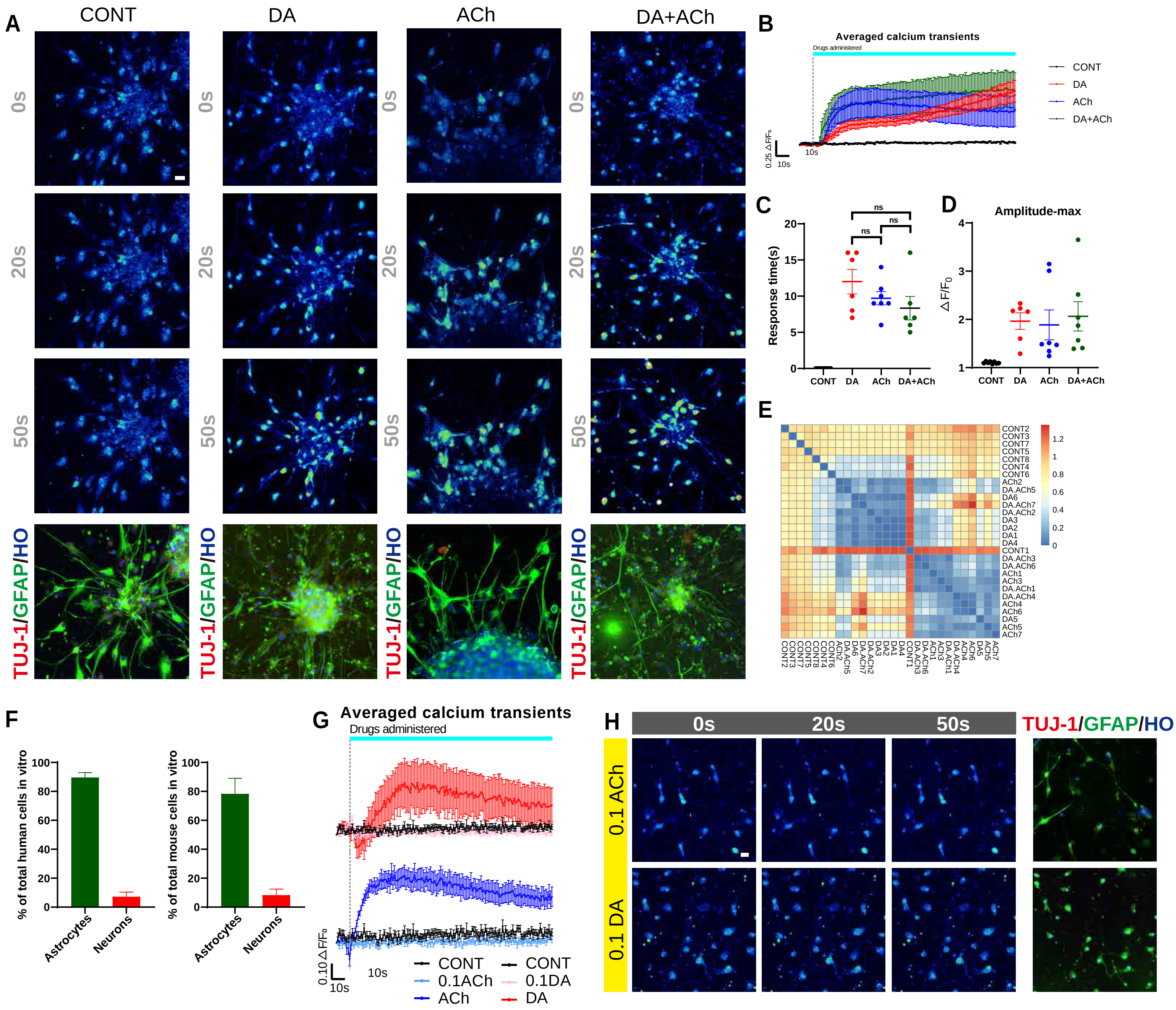

Supplementary Figure 6

Figure S6 Mouse and Human Astrocytes Cultures Treated with Neurotransmitters

(A) Representative snapshots of calcium activity in primary mouse astrocytes after neurotransmitters treatment.

(B) Average calcium transients of astrocytes in each group in A ($n \geqslant 6$ cells for each group).

(C and D) Quantification of response time and maximum amplitude of calcium transients in mouse astrocytes ($n \geq 6$ cells for each group).

(E) Correlation of calcium transients of each astrocyte in four groups in A.

(F) Quantification of proportion of astrocytes among the human-derived and mouse-derived cells *in vitro* ($n = 6$ replicates).

(G and H) Representative calcium activity of human astrocytes, with addition of 0.1 μm DA or ACH ($n = 8$ cells for each group).

Data are represented as mean ± SEM; scale bars: 20 μm. two-sided Student's t-test in C, ns: $p < 0.05$.

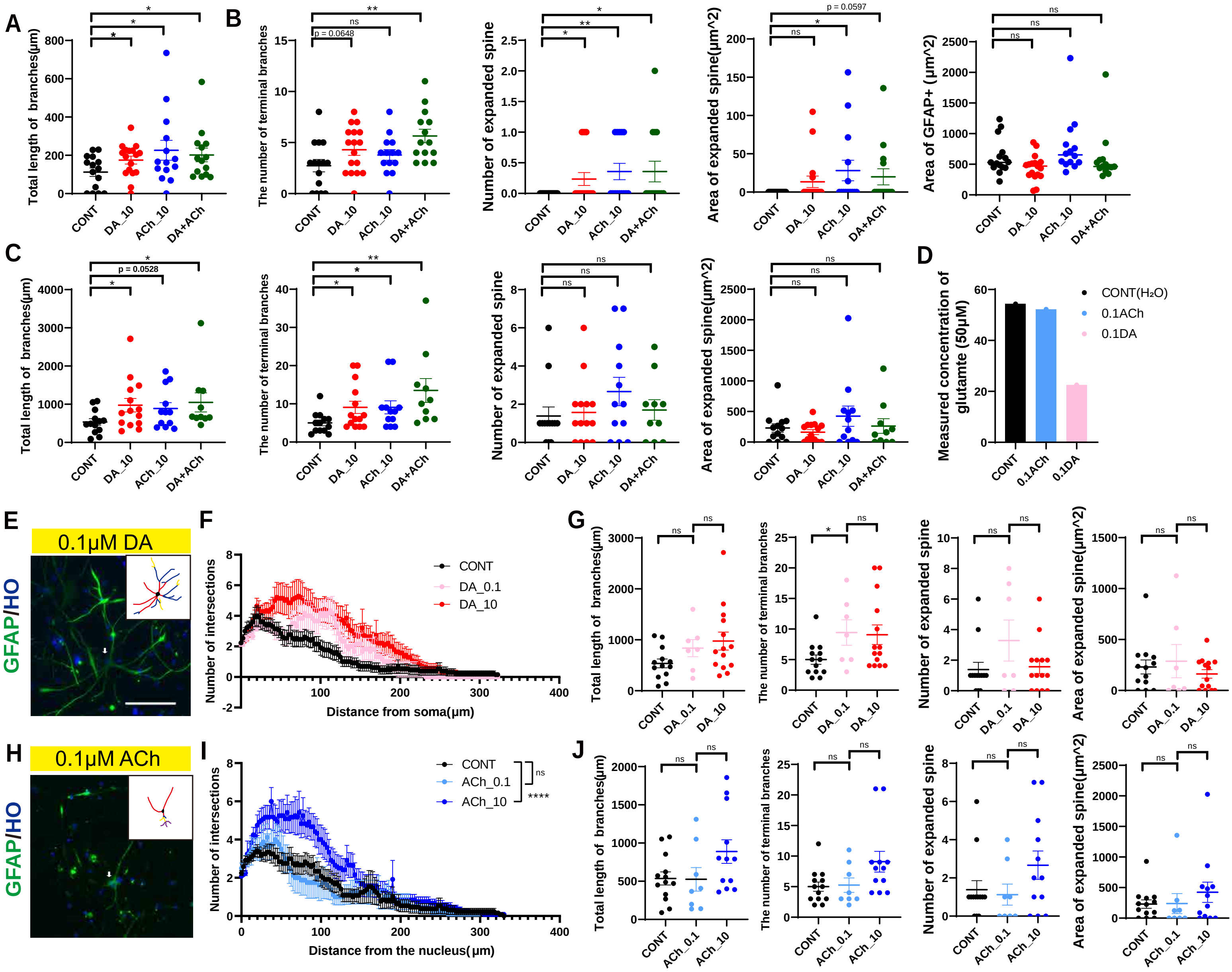

Supplementary Figure 7

Figure S7 Morphological complexity of Astrocytes Cells Increased after Neurotransmitter Treatment *In Vitro*.

(A and B) Quantification of morphological results of human astrocytes in Figure 7J ($n = 15$ cells for each group).

(C) Quantification of morphology of mouse astrocytes in Figure 7L ($n \geq 10$ cells for each group).

(D) The actually measured value for 50 μM glutamate after adding neurotransmitters (ACh, DA). Group DA was excluded from the test, as the dissolved black substance appeared after administration, which led to dysfunction of the kit.

(E-J) Representative images and quantification of morphology of primary mouse astrocytes (some data from the test in Figure 7L) after neurotransmitter (0.1 mM) treatment ($n \geq 6$ cells for each group).Data are represented as mean ± SEM; scale bar: 100 μm; two-way ANOVA in I, two-sided Student's t-test in A, B, C, G, and J, $*p < 0.05$, $**p < 0.01$, $***p < 0.001$, $****p < 0.0001$.
