# Supplementary material for: Single‐Cell Transcriptome Landscape and Cell Fate Decoding in Human Brain Organoids after Transplantation
Source: Adv Sci (Weinh). 2024 May 6;11(28):2402287. doi: 10.1002/advs.202402287 (PMC11267311; doi:10.1002/advs.202402287)
Supplement: Supplementary file 2 — Supporting Information [file ADVS-11-2402287-s002.docx]

| REAGENT or RESOURCE | SOURCE | IDENTIFIER |
| --- | --- | --- |
| **Antibodies** | | |
| Mouse anti-Human Nuclei (hN) [1:500] | Chemicon | MAB1281 |
| Rabbit anti-Ki67 [1:500] | ZYMED | 180191Z |
| Mouse anti-NeuN [1:500] | Arigo | ARG53383 |
| Goat anti-SOX2 [1:500] | R&D | AF2018 |
| Mouse anti-PKC-λ [1:500] | BD | 610207 |
| Rabbit anti-PAX6 [1:1000] | Covance | PRB-278P |
| Mouse anti-β-III Tubulin [1:1000] | Sigma-Aldrich | T866 |
| Rabbit anti-β-III Tubulin [1:1000] | Covance | PRB-435P |
| Rabbit anti-TBR1 [1:1000] | Abcam | AB31940 |
| Mouse anti-BRN2 [1:300] | Santa Cruz | sc-393324 |
| Rabbit anti-GFAP [1:1000] | Dako | Z0334 |
| Mouse anti-GFAP [1:500] | Sigma-Aldrich | MAB360 |
| Mouse anti-NCAM [1:500] | Santa Cruz | SC-10 |
| Mouse anti-Human  Synaptophysin (hSYN) [1:500] | eBioscience | 147-6525-80 |
| Goat anti-NESTIN [1:1000] | Santa Cruz | sc-21247 |
| Goat anti-NANOG [1:200] | R&D | AF1997 |
| Rat anti-CD31 [1:200] | Biolegend | 102501 |
| Rabbit anti-PDGFra [1:500] | Cell Signal Technology | 3174s |
| Mouse anti-STEM121 [1:500] | Stem Cell | AB-121-U-050 |
| Rabbit anti-MBP [1:500] | Sigma-Aldrich | AB5864 |
| Rat anti-CTIP2 [1:100] | Abcam | ab1845 |
| Mouse anti-MAP2 [1:1000] | Sigma-Aldrich | M1406 |
| Rabbit anti-DCX [1:500] | Cell Signal Technology | 4604 |
| Rabbit anti-TH [1:500] | Pel-Freez | P40101 |
| Rabbit anti-VACHT [1:500] | Synaptic Systems | 139103 |
| Mouse anti-STEM123 [1:500] | Stemcells | AB-123-U-050 |
| **Chemicals, peptides, and recombinant proteins** | | |
| SB431542 | Tocris | 301836-41-9 |
| DMH-1 | Tocris | 1206711-16-1 |
| bFGF | Gibco | PHG0263 |
| hEGF | Gibco | PHG0311 |
| N-2 supplement (100x) | Gibco | 17502048 |
| B-27 supplement (50x) | Gibco | 17504044 |
| Matrigel | Corning | 356234 |
| VTN | Gibco | A14700 |
| Hoechst | Invitrogen | H1398 |
| Fluoromount-G | SouthernBiotech | 0100-01 |
| MEM Non-Essential Amino Acids Solution (100X) | Gibco | 11140 |
| DMEM/F-12 | Gibco | 8117262 |
| TrypLE | ThermoFisher Scientific | 12604013 |
| Dispase | Gibco | 17105041 |
| Fetal Bovine Serum (FBS) | Life Technologies | 10099-141 |
| Essential 8 Medium | Life Technologies | A14666SA |
| ROCK inhibitor (Y-27632) | Stem Cell | 72304 |
| Dopamine hydrochloride (DA) | Sigma-Aldrich | 1225204 |
| Acetylcholine chloride (ACh) | Sigma-Aldrich | A2661 |
| **Critical commercial assays** | | |
| Chromium Single Cell 3′ v3 Library kit | 10X Genomics | PN-1000075 |
| Rhod-4 calcium imaging kit | Abcam | ab112157 |
| Glutamate Assay Kit | Sigma | Mak004 |
| **Deposited data** | | |
| Single-cell RNA sequencing data | This paper | NCBI GEO: GSE243015 |
| **Experimental models: Cell lines** | | |
| H9: AAVS-ChR2-EYFP | This paper | N/A |
| H9 | WiCell | 16-W0060 |
| IMR 90-4 | WiCell | 17-W0063 |
| **Software and algorithms** | | |
| GraphPad Prism | GraphPad Software | Version 8.3.0 |
| R | Posit, Joseph J. Allaire | Version 4.0.5 |
| Cell Ranger | 10X Genomics | Version 3.1 |
| ImageJ | National Institutes of Health | Version 1.53C |
